# Proportional Changes in Cognitive Subdomains During Normal Brain Aging

**DOI:** 10.3389/fnagi.2021.673469

**Published:** 2021-11-15

**Authors:** Yauhen Statsenko, Tetiana Habuza, Klaus Neidl-Van Gorkom, Nazar Zaki, Taleb M. Almansoori, Fatmah Al Zahmi, Milos R. Ljubisavljevic, Maroua Belghali

**Affiliations:** ^1^Department of Radiology, College of Medicine and Health Sciences, United Arab Emirates University, Al Ain, United Arab Emirates; ^2^Big Data Analytics Center, United Arab Emirates University, Al Ain, United Arab Emirates; ^3^Department of Computer Science and Software Engineering, College of Information Technology, United Arab Emirates University, Al Ain, United Arab Emirates; ^4^Department of Neurology, Mediclinic Middle East Parkview Hospital, Dubai, United Arab Emirates; ^5^Department of Clinical Science, Mohammed Bin Rashid University of Medicine and Health Sciences, Dubai, United Arab Emirates; ^6^College of Education, United Arab Emirates University, Al Ain, United Arab Emirates

**Keywords:** machine learning, cognitive domains, cognitive decline, aging, clinical psychology, neurodegeneration, proportionality, psychophysiological test

## Abstract

**Background:** Neuroscience lacks a reliable method of screening the early stages of dementia.

**Objective:** To improve the diagnostics of age-related cognitive functions by developing insight into the proportionality of age-related changes in cognitive subdomains.

**Materials and Methods:** We composed a battery of psychophysiological tests and collected an open-access psychophysiological outcomes of brain atrophy (POBA) dataset by testing individuals without dementia. To extend the utility of machine learning (ML) classification in cognitive studies, we proposed estimates of the disproportional changes in cognitive functions: an index of simple reaction time to decision-making time (ISD), ISD with the accuracy performance (ISDA), and an index of performance in simple and complex visual-motor reaction with account for accuracy (ISCA). Studying the distribution of the values of the indices over age allowed us to verify whether diverse cognitive functions decline equally throughout life or there is a divergence in age-related cognitive changes.

**Results:** Unsupervised ML clustering shows that the optimal number of homogeneous age groups is four. The sample is segregated into the following age-groups: Adolescents ∈ [0, 20), Young adults ∈ [20, 40), Midlife adults ∈ [40, 60) and Older adults ≥60 year of age. For ISD, ISDA, and ISCA values, only the median of the Adolescents group is different from that of the other three age-groups sharing a similar distribution pattern (*p* > 0.01). After neurodevelopment and maturation, the indices preserve almost constant values with a slight trend toward functional decline. The reaction to a moving object (RMO) test results (RMO_mean) follow another tendency. The Midlife adults group's median significantly differs from the remaining three age subsamples (*p* < 0.01). No general trend in age-related changes of this dependent variable is observed. For all the data (ISD, ISDA, ISCA, and RMO_mean), Levene's test reveals no significant changes of the variances in age-groups (*p* > 0.05). Homoscedasticity also supports our assumption about a linear dependency between the observed features and age.

**Conclusion:** In healthy brain aging, there are proportional age-related changes in the time estimates of information processing speed and inhibitory control in task switching. Future studies should test patients with dementia to determine whether the changes of the aforementioned indicators follow different patterns.

## 1. Introduction

Neuroscience lacks a reliable means for screening patients in the early stages of dementia (Habuza et al., 2021a,d). Furthermore, the accuracy of clinical diagnostics of the disease is also limited (see Table 1). This may delay the identification of dementia for over a year, thus reducing the supposed benefits of early treatment (e.g., an improvement of memory, reduction of anxiety, and engagement into social activities) (Milne, 2010).

**Table 1:** Accuracy and limitation of methods of diagnosing dementia.

**Method**		**Diseases studied**				**Limitations**
			**Sensitivity,%**	**Specificity,%**	**References**	
MRI		MCI to AD progression^*^	87	66	Desikan et al., 2010	In either case, it is often important to not appear to be overly certain, as in most instances imaging features are not pathognomonic Gaillard, 2021
		Parkinson's Disease	61	68	Nemmi et al., 2019		
		Multiple sclerosis	93	81	Eitel et al., 2019		
Tractography		Amnestic MCI	96	94.2	Jung et al., 2018		
		Parkinson's disease	40–86	41–94	Kamagata et al., 2013		
fMRI		mild AD	77.3	70	Balthazar et al., 2014	Altered BOLD signals found in AD/MCI patients can reflect impairments in haemodynamic processes separately from changes in neuronal activity Göttler et al., 2019
PET		AD vs. FTLD	69.4	93.2	Kim et al., 2019	Despite promising reliability and accuracy, PET/MRI exists predominantly as a research-based tool in dementia as it requires specific radiotracers Lorking et al., 2021
		AD vs. MCI	81.8	86	Arbizu et al., 2013		
		Amyotrophic lateral sclerosis	94.8	80	Van Laere et al., 2014		
Angiography		Dementia with Lewy bodies (DLB)	93	87	Kamagata et al., 2017	The technique exposes the patient to radiation as well as iodinated contrast which may induce nephropathy and allergic reactions
Brain perfusion	MRI	AD vs. FTLD	69	68	Steketee et al., 2016	Damage of the brain function in FTLD, assessed with ASL perfusion, can vary regionally despite widespread atrophy Shimizu et al., 2010
	SPECT	Dementia	61	70	Uchida et al., 2006		
		DLB vs. AD	87–100	90–96	Roquet et al., 2016		
	SPECT + MMSE	DLB vs. AD	81	85	Hanyu et al., 2006		
Cognitive tests	FBI	FTLD	90	100	Kertesz et al., 2003	The tests may exhibit the limited sensitivity to subtle brain abnormalities Spencer et al., 2013. The results should be carefully transfered from one to another type of cognitive impairment Freitas et al., 2012
	Mini-Cog	Cognitive Impairment	60	90	Carnero-Pardo et al., 2013		
	MoCA	Vascular dementia	77 vs. 85	97 vs. 88	Freitas et al., 2012		
	MMSE	MCI vs. dementia	88 vs. 84	70 vs. 86	Tsai et al., 2016		
	full vs. short MoCA	88 vs. 79	74 vs. 80	Tsai et al., 2016		

* *AD, Alzheimer's disease; BOLD, blood-oxygen-level-dependent; DLB, Dementia with Lewy bodies; FBI, Frontal Behavioral Inventory; FTLD, fronto-temporal lobe dementia; MCI, mild cognitive impairment; MMSE, Mini Mental State Examination; MoCA, Montreal Cognitive Assessment*.

There are several ways to improve the diagnostics. The first one is *a multi-modal approach to identify early dementia*. The second approach is *an informant-based assessment* during the primary diagnostics. Scientists and medical professionals advocate for this because good knowledge of the personality of the patient is crucial for the disease identification. The informant-based assessment is reported to be more reliable than the Mini-mental state examination (MMSE) (Panegyres et al., 2016). The third approach is *a further development of the screening strategy*. To be trusted and widely used in practice, a novel test should be based on the concepts of brain aging. The prevailing solution comprises three steps: 1) investigating possible new causes of brain aging; 2) testing the reliability of the test in a healthy population; and 3) testing the reliability in patients with dementia.

Due to the growing interest in cognitive changes in the aging brain, researchers have accumulated facts that provide segmental insight on changes in reaction speed, working memory (Chai et al., 2018; Verhaeghen, 2018), executive functions (Rosado-Artalejo et al., 2017; Nyongesa et al., 2019), memory, linguistic abilities, and knowledge (Antoniou and Wright, 2017; Peelle, 2019). To describe the mechanism of cognitive decline with advancing age, scientists introduced a concept of *brain reserve* and *cognitive reserve* both mitigating consequences of traumas to the head, aging, and neurodegenerative diseases (Medaglia et al., 2017). However, a general theory that would meet all the needs of medical practitioners is missing. The following questions remain unaddressed.

First, both cognitive and brain reserves are not well defined yet. The concept of reserve relies on anatomic measurements (the cranial volume, and its height and length) as predictors of the brain reserve (Brickman et al., 2011). According to novel findings, the list of predictors should be extended to the total count of neurons, synapses, and dendrites in the brain (Cullati et al., 2018). In contrast to the aforementioned structural findings, the cognitive reserve comprises a set of psychological factors and different lifestyle activities throughout life (Brickman et al., 2011). However, there is an opinion that the cognitive reserve consists of the neural reserve and neural compensation (Lee et al., 2019).

Second, it is not clear what exactly accounts for the resilience of the brain to cognitive decline. There is an opinion that the brain reserve is a primary defense, and it defines a potential of the cognitive reserve (Van Loenhoud et al., 2018). People with more neurons may face dementia later compared to people with a lesser brain reserve (Giovacchini et al., 2019). Contrarily, neuroplasticity depends on premorbid intelligence, education, and lifestyle (cognitive reserve), e.g., people with more years of education and higher intelligence may cope better with dementia.

Moreover, cognitive and brain reserves are independent in moderating symptoms of dementia (Groot et al., 2018). Further research is required to study a relationship between the brain reserve and the cognitive reserve.

To date, psychophysiological tests widely used for assessing cognitive domains have not been fully studied or justified in such aspects as accuracy and implication. As an example, a meta-analysis of cognitive tests showed that MMSE—the most commonly used tool—had the lowest sensitivity for diagnostics of Mild Cognitive Impairment (MCI) (Breton et al., 2019). This was confirmed by another review that also showed that the predictive power of MMSE and Montreal Cognitive Assessment (MoCA) is more limited than in recall tests (Tsoi et al., 2017).

### 1.1. Significance of Studies on Cognitive Decline in Healthy Aging

#### 1.1.1. Novelty of Studies on Age-Related Changes in Cognitive Subdomains

Though some studies are dedicated to comparison of patients with neurodegenerative diseases with cognitively normal population (Nemmi et al., 2019; Rehman et al., 2019), they have limited value as the processes that unfold in normal aging are not studied well (Boyle et al., 2013; Murman, 2015; Hedden et al., 2016). There is no strong concern about the pathophysiological changes responsible for aging. Age-related cognitive decline seems to be conciliated by underlying neurobiological changes, such as vascular changes and accumulation of neuropathology (Hassenstab et al., 2016; Statsenko et al., 2021b,c). The growing support for an alternative hypothesis that estimates cognitive change in older adults may have been biased negatively by the influence of diseases that are common in late life, especially neurodegenerative diseases such as Alzheimer's disease (Spiro III and Brady, 2011).

Studies on accelerated brain aging are of limited value without knowledge of what can be considered as normal cognitive changes (Kaufman et al., 2016). It is quite evident that all these studies have common limitations and misclassification bias. Most research found it difficult to differentiate pathological cognitive declines from normal cognitive aging especially among adults over the age of 65 years old (Rönnlund et al., 2005; Salthouse, 2016, 2019).

There is a large number of neurodegenerative diseases and schemes of classifying them (Armstrong, 2012), e.g., a classification based on neuropathological findings provides over 10 groups with many nosologies within each group (Kovacs, 2019). Researchers struggle to count and put all these diseases into a system because it is a huge number to cover. A clinical appearance along with non-invasive diagnostic signs is not specific enough to distinguish these diseases. This requires invasive neuropathological examination (Kovacs, 2019). In such circumstances, one cannot cover the whole range of neurodegenerative diseases with comparative studies. This could explain why some researchers try to work out a model of normal aging with non-invasive studies, e.g., cognitive test and MRI (Salthouse et al., 2003; Tamnes et al., 2013; Storsve et al., 2014; Viviano et al., 2017; Chen et al., 2018; Salthouse, 2019; Habuza et al., 2021b; Statsenko et al., 2021d). These researchers of brain aging do not try to compare healthy adults with the individuals that suffer from neurodegeneration. Instead of concentrating on how to identify a particular disease, they tend to highlight the permissible changes in the healthy population. This helps to raise suspicion of the accelerated brain aging in the outstanding cases. We use a similar approach in our studies (Gorkom et al., 2021; Statsenko et al., 2021e,f; Uzianbaeva et al., 2021).

#### 1.1.2. Actuality of Studies on Information Processing

*Age-related changes in information processing speed* are an issue of ongoing studies (Hong et al., 2015; Finkel et al., 2016; Adólfsdóttir et al., 2017). An exceptional interest in this cognitive feature is based on the fact that changes in information processing speed correlate with functional abilities in older adults (Wahl et al., 2010; Finkel et al., 2016). Many research questions in these studies are not covered yet. Recent studies on the issue were dedicated to structural and biochemical correlates of changes in processing information. For example, a study of typical aging reported an inverse relationship between information processing speed and both the interleukin-6 level and fractional anisotropy of corpus callosum (Bettcher et al., 2014). Another study highlighted correlations between larger volume of corpus callosum, lower levels of the inflammatory marker and insulin, and greater self-reported physical activity of intact older adults (Bott et al., 2017).

Some associate age-related decline in cognitive speed with white matter integrity (Kerchner et al., 2012; Salami et al., 2012; Hong et al., 2015). However, it is not entirely clear what are the main factors responsible for white matter changes. There is still no sufficient explanation for neurocognitive slowing observed in aging.

By doing such research, scientists try to find out the reasons for changes in the information processing speed during normal aging. If studied thoroughly in a healthy population, the reasons can be compared with the ones in patients with neurodegeneration. This will evidence risk factors, e.g., genetics (Cheng et al., 2014) and white matter changes (Hayes, 2017). In such a way, studies of a healthy population may contribute to identification and differentiation of the early signs of neurodegenerative diseases in the future.

There are discrepant findings regarding age-related changes in information processing speed depending on the tasks used. Younger adults performed better in a symbol search and coding task, whereas older adults were more proficient in inspection time task (Ebaid et al., 2017). There is no convincing explanation what accounts for this. In another study with the same task, the performance speed slowed down across the lifespan (Ebaid and Crewther, 2019).

Interestingly, some researchers claim that a decline in sensory function reduces the processing speed (Tam et al., 2015; Ji et al., 2019). The results of the studies indicated that cognitive processing speed predicted general cognitive status in older but not in younger adults. Future research may be needed to verify the findings (Statsenko et al., 2021d).

#### 1.1.3. Actuality of Research on Inhibitory Control and Task Switching

Task switching has commonly attracted a particular interest of neurophysiologists as it accounts for proficiency in multitasking. Inhibition of planned response actions is used to explain various findings in such studies. It is a known fact that seniors experience difficulties in sustaining attention and inhibiting behavioral responses to the stimuli that are inconsistent with the selected goals. Despite this, studies on inhibitory control during normal aging remain actual as distinctive features and exact mechanisms of such a deterioration are not well justified (Gade et al., 2014; Kray and Ferdinand, 2014; Gaál and Czigler, 2015, 2017).

Since some studies managed to establish a relationship between white matter integrity and inhibitory control (Ystad et al., 2011; Wolf et al., 2014; Hayes, 2017; Li et al., 2018) there is an assumption that age-related changes in the white matter underlie inhibitory control deficits in the elderly. Nevertheless, specific evidence for this from a structural neuroscience perspective is lacking (Coxon et al., 2012). Some researchers investigated lifespan trajectories of inhibition deficit. The results hint to a qualitative change of task switching at the age of 60 years old. However, the neuronal reasons for this are poorly understood (Nyberg et al., 2012; Van der Elst et al., 2013; Adólfsdóttir et al., 2017).

In the last decade, a large number of studies tested the hypothesis that older adults experience inhibition deficit (Pettigrew and Martin, 2014; Gade et al., 2017; Hsieh and Lin, 2017). Surprisingly, some studies with both cross-sectional (Pettigrew and Martin, 2014; Hsieh and Lin, 2017) and longitudinal design (Adólfsdóttir et al., 2017) revealed the presence of age-related decline in inhibitory functioning while another study did not verify it (Sebastian et al., 2013). A difference in the tasks and strictness of inclusion criteria may resolve the controversy. To prove this assumption, new studies should be initiated.

There are two distinct types of inhibition that are based on two dissociable processes: the threshold adjustment process involving the global inhibition of motor output and the controlled selection process involving competitive inhibition among coactive responses. Recent findings show that the threshold adjustment process functions differently in early and late adulthood (Larson et al., 2016; Erb et al., 2020). There is a hypothesis that cognitive fatigue due to the tasks provided (e.g., Flanker task) may impact the findings in older adults and mislead the research.

### 1.2. Processes Underlying Cognitive Changes

Several processes may account for the declines associated with cognitive aging (Zelinski et al., 2011). Two of them, atrophy and neuroplasticity, are in direct theoretical opposition. The concept of brain atrophy is intrinsically associated with vascular factors (Borja et al., 2020) and inflammation (Alkasir et al., 2017), dietary habits (Seetharaman, 2016), metabolic disorders (Komuro et al., 2020), and dysbiosis (Alkasir et al., 2017). These factors provide important clues to dementia-related mechanisms and support the concepts regarding the management of cognitive impairment through the modulation of these factors. In contrast, the concept of brain neuroplasticity suggests that behaviors such as aerobic exercise and direct cognitive training can serve as compensatory factors that may potentially reduce the appearance of cognitive aging and hinder the signs of dementia (Zelinski et al., 2011).

### 1.3. Concurrent Changes in Cognitive Domains During Aging

Declines vary by cognitive domain. Many of them are subject to deterioration, whereas others, such as language, remain stable. In general, age-related changes in reaction speed underlie changes in a number of cognitive domains (Salthouse et al., 2003). The decline in information processing affects executive functioning (EF), a cognitive domain that accounts for individual goal-directed behavior. This may be explained in two ways. The first is that relevant operations cannot be executed within the required (limited) period. The second is that slow processing reduces the amount of simultaneously available information and that the higher-level processing shuts down without the information supply (Salthouse, 1996). For this reason, EF deficit has the potential to affect performance in a wide variety of cognitive variables and may serve as a potential mediator of age-related cognitive decline (Salthouse et al., 2003).

Studies suggest that there are several interdependent cognitive aging mechanisms. Despite the common pattern of findings for different speed-based tasks, cognitive slowing is not known to be a general factor of decline in abilities (Zelinski et al., 2011; Anderson and Craik, 2017). For example, working memory is required for recalling a task. It correlates positively with reasoning. Logically, its impairment has substantial ramifications for cognitive performance in older adults.

**Executive Functioning (EF) and Cognitive Control**. *Cognitive control* is the ability to orchestrate our thoughts and actions per internal goals. EF is commonly used as an umbrella term that covers both EF and cognitive control and applies to a set of higher-order (cognitive) processes involved in organizing intentional behavior in a novel situation (Miyake and Friedman, 2012). EF comprises three subdomains: *inhibitory control* that involves the preclusion of irrelevant information and the prohibition of prepotent responses; *task switching* that is the ability to switch flexibly between mental sets; and *updating* that is the constant monitoring and rapid addition/deletion of working memory contents. Decreased inhibitory control is a feature of age-related cognitive decline (Maldonado et al., 2020). Switching between different tasks requires working memory if a cue is absent, and a decline of the memory domain across time may impact this EF (Aschenbrenner and Balota, 2015; Moreira et al., 2018).

**Attention** is typically divided into two global subdomains: selective attention (e.g., concentration) and sustained attention or vigilance (e.g., divided attention) (Harvey, 2019). The elderly have a reduced ability to pay attention to selected stimuli. Age-related declines in selective attention interact with other cognitive domains and other known age-related changes (Zanto and Gazzaley, 2017).

There are other attentional subsystems. For example, it was shown that aging affects the balance between goal-guided and habitual spatial attention (Twedell et al., 2017). By the habitual spatial attention, authors of this study understand attention to the target that attracts notice more frequently, i.e., a space with the high-probability of triggering stimuli. Older adults suffer from disrupted goal-guided attention, but they are relatively unimpaired in deploying spatial attention through incidental habit-based learning. Hence, training older adults to develop good search habits may help compensate for a decline in some other attentional subdomains. All the attentional skills have EF components, the age-related decline of which impacts attention (Harvey, 2019). Older adults have a deficit in inhibiting irrelevant information, presumably because of changes in the prefrontal cortex (Zanto and Gazzaley, 2017).

**Memory**, as a cognitive domain, may be affected by multiple processes including speed, working memory, executive control operations, and sensory declines. Memory declines in aging may stem from a deficient ability to dynamically allocate attention and switch between functional brain networks. Because of structural and functional brain changes, older adults do not ignore distraction but, rather, co-encode relevant and irrelevant information. This leads to the overload of the limited cognitive resources (Zanto and Gazzaley, 2017).

Despite the well-established fact that memory performance declines with age, not all aspects of memory are impaired equally (Balota et al., 2000). *The procedural memory* is manifested without the direct recollection of the previous events, whereas *the declarative memory* is revealed through intentional retrieval of previous experience. It encompasses the episodic memories on some events and the *semantic memories* that reflect our general knowledge of facts and word meanings. In general, older adults have the most significant memory deficits that appear in long-term episodic memory because of the major attention demand. The semantic, sensory, and procedural memory have a minimal demand for attention, and they produce relatively little age-related change in performance (Balota et al., 2000; Zanto and Gazzaley, 2017).

The mediation of memory with other cognitive domains is described with the concept that semantic memory is an organized data storage of words or concepts - “nodes” - connected to other nodes *via* associative pathways. When an individual by directing attention activates a node, the activation spreads through the network from the node to other related nodes for subsequent processing (Balota et al., 2000).

**Working memory** as a temporary buffer for cognitive processing undergoes such age-related changes as a reduction of its capacity and speed of processing. The reasons for the changes are a decline in the processing speed and/or a breakdown in the basic control processes (resistance to interference, task coordination, memory updating, binding, and/or top-down control as inferred from neuroimaging data). Working memory is more age-sensitive regarding spatial rather than verbal material (Verhaeghen, 2018).

**The age-related sensory and perceptual deficits** exacerbate cognitive decline and complicate the performance of cognitive tasks. Declines in these domains indicate a reduction of the ability to detect a stimulus that occurs in one of the five sensory modalities, to process and integrate the acquired information. Clinically, the elderly may experience a variety of challenges in the identification of objects, sounds, tastes, smells, and tactile sensations. These include *agnosia*, the inability to recognize previously identifiable objects (Harvey, 2019). Cognitive aging in sensation and perception domains impacts other domains in turn. For example, at any age, hearing deficits lead to auditory memory problems and may affect language comprehension (Zelinski et al., 2011). There are strategies to compensate for declines in perceptual processes. The “posterior-to-anterior shift in aging” model by Davis describes the increased prefrontal activity as a means to overcome declines in perceptual processes that occur in occipital regions. This accounts for the task performance comparable in both older and younger adults, although it utilizes different neural mechanisms (Zanto and Gazzaley, 2017).

**Motor skills** (the basic elements of motor activity) and *construction* (the ability to either copy or produce drawings of common objects, as in clock drawing paradigms) may be impaired in severe cases of dementia, damage to the nondominant hemisphere, or lesions to the parietal cortex (Harvey, 2019).

**Language.** Some cognitive processes associated with language remain stable or improve into the mid-70s (Zelinski et al., 2011). Language deficits may be associated with a deficit in EF (e.g., the ability to access semantic storage successfully) or with a slowed processing speed (Harvey, 2019).

A general slowness of a neurologic response is the major factor in comprehension impairment. Additionally, attentional changes and memory decline impact speech comprehension (Obler and Albert, 1981).

**Inseparability and intercorrelation of cognitive domains**. Cognitive domains should not be viewed as lacking validity if they are intercorrelated. There is considerable evidence that in many patient populations, including those with schizophrenia and bipolar disorder, conventional domains of cognitive dysfunction are not truly separable (Harvey, 2019).

## 2. Objectives

We aim to improve the diagnostics of age-related cognitive functions by developing insight into age-related changes in cognitive subdomains. Specifically, we want to determine whether diverse executive functions decline proportionally throughout life.

*Hypothetically*, cognitive domains are interdependent, and the pace of their age-related decline is thought to be approximately equal. Moreover, there is a concept that explains the normal neurocognitive slowing with the slowing of central or computational processing common for all cognitive functions. Alternatively, disproportional changes may indicate accelerated brain aging.

To address the objectives, we formulated the following tasks:

1. To develop new indices that reflect the ratio of cognitive functional activities during psychophysiological task performance.
2. To divide the studied sample into an optimal number of age-groups with regard to the psychophysiological test results, and find attributes that can be used as subtle biomarkers of age-group identification by assessing the performance of the deployed unsupervised ML model.
3. To study the distribution of the novel designed indices and selected psychophysiological attribute values across the lifespan in both sexes.
4. To inspect possible associations of the age with the newly proposed scores, and to determine the predictive potential of PTs to identify the values of the indices developed.

## 3. Materials and Methods

### 3.1. Psychophysiological Tests Used

To estimate the psychophysiological status, we used a battery of neurophysiological tests that involve such cognitive domains and subdomains as attention, working memory, information processing speed, task switching and inhibitory control, and executive control (Statsenko and Charykova, 2010; Statsenko et al., 2019, 2020). The tests and their dependent output variables are listed below.

**Figure 1:**
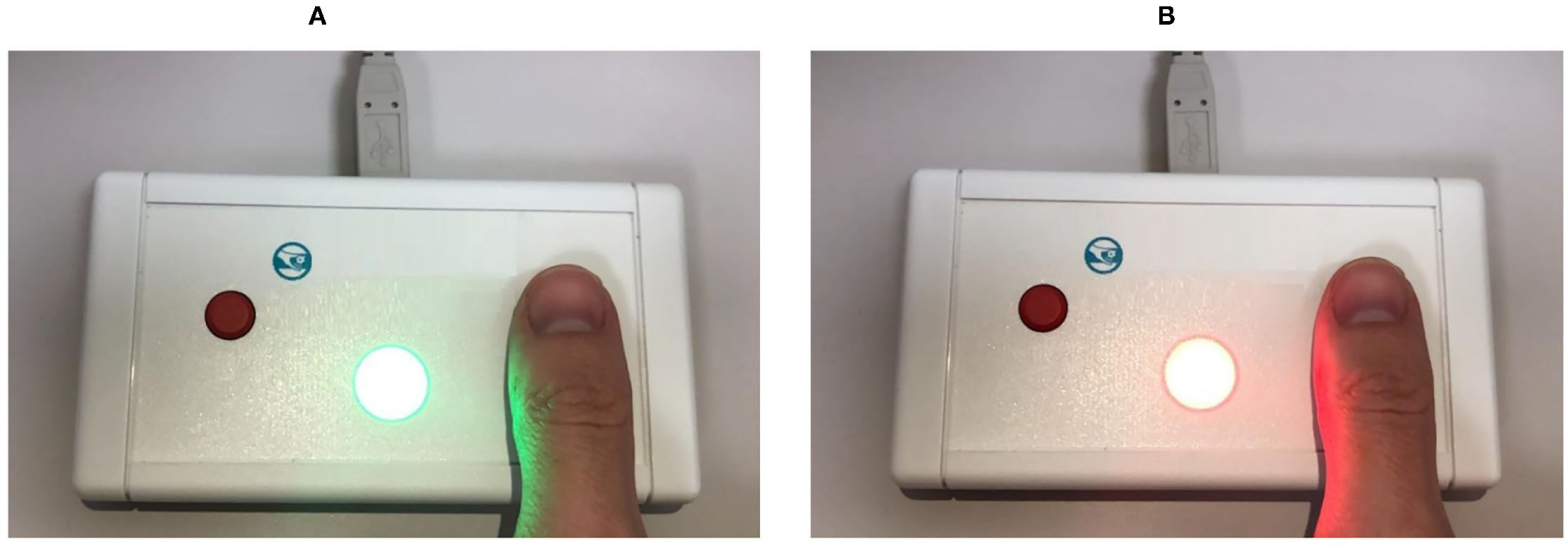
Simple and complex visual-motor reaction test (for details see Subsection 3.1).

**Figure 2:**
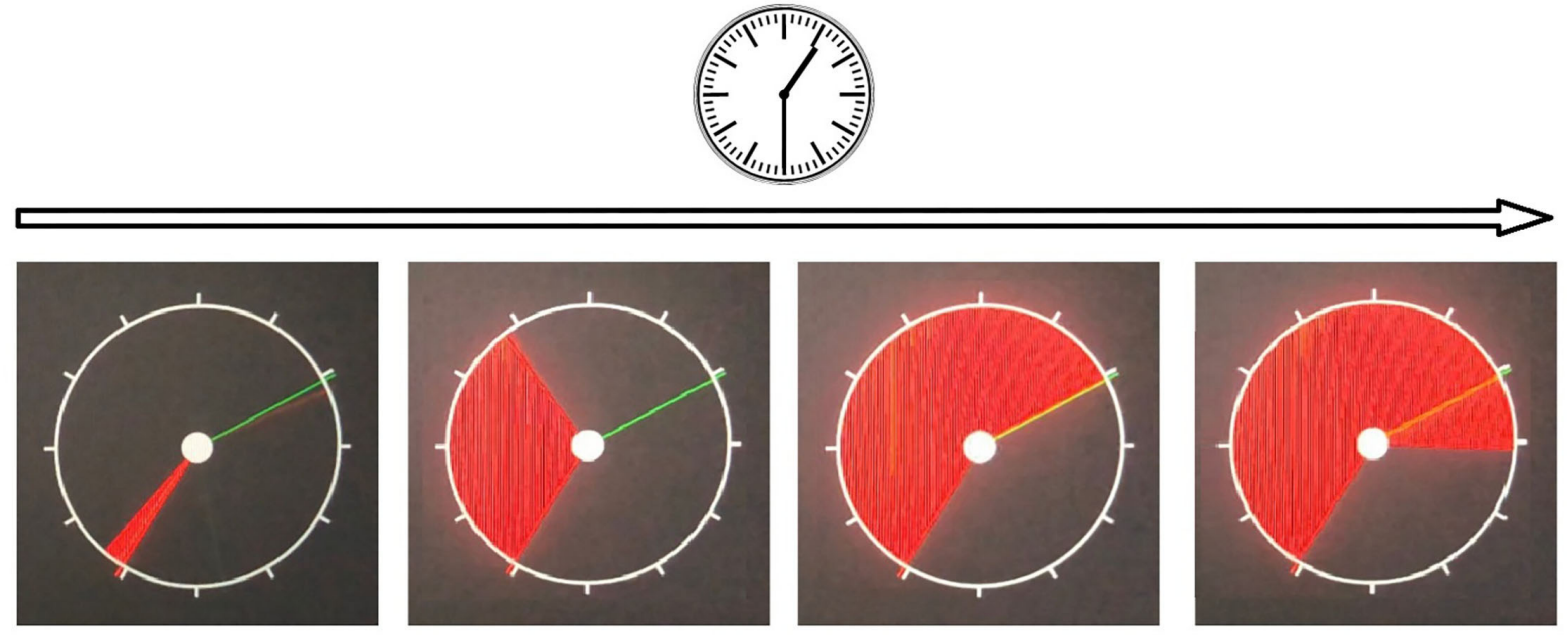
Reaction to a moving object test.

**Figure 3:**
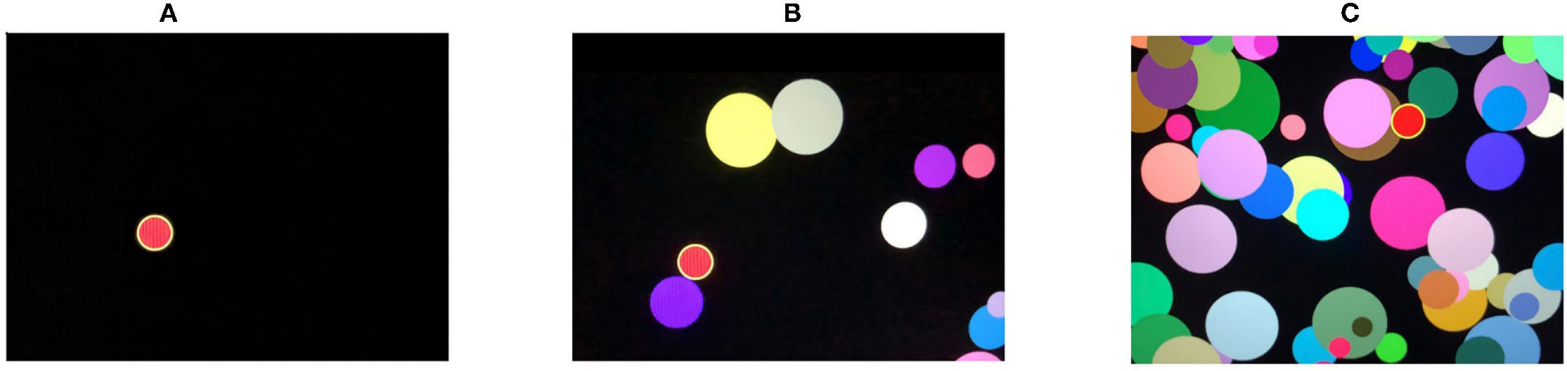
Attention study and interference resilience technique (for details see Subsection 3.1).


1. *Simple visual-motor reaction (SVMR)*. The task estimates mean reaction time recorded as a result of subsequent attempts in the task with the only type of stimuli and the only way to respond to them. Reaction time (ms) is a major dependent variable. Typically, the test contains over 30 trials (SVMR_trialsNo) with unequal intervals of time between them. One can calculate the mean value of reaction time out of subsequent episodes of testing (SVMR_mean). By making a set of trials, researchers improve the accuracy of assessing information speed processing. They also count the number of mistakes made by the examinee (SVMR_mistakes). The mistakes can be either missing the targeted events (SVMR_passes) or preliminary responding (SVMR_falstart).
  SVMR time reflects the mobility of the examinee. The mean reaction time under 177 ms accounts for the pronounced mobility of the nervous processes. The range of values from 177 to 200 ms is characteristic of a mobile type of the processes. Its length within 200–210 ms depicts an average type of the nervous processes. SVMR_mean value of 210–233 ms indicates the inertial status of the nervous system. After reaching the threshold value of 233 ms, the pronounced inertia is diagnosed. The SD (SVMR_variance) is a measurement that shows how the length of the reaction is scattered in time.
2. *Complex visual-motor reaction (CVMR)* is a variant of the “go/no-go” test in which the examinee is to respond to one of the two possible types of triggering stimuli. We asked the examinee to respond by pushing the button when the green indicator light appears (see Figure 1A). In reverse to this condition, when the indicator is colored red, no motor response is expected (see Figure 1B). The system records the latency between the time the light emerges and the time the examinee responds. The result is processed as mean length of response time calculated after 30 subsequent presentations of the triggering stimulus (CVMR_mean). We also reported a number of mistakes (CVMR_mistakes). Those were the mistakes analagous to SVMR: missing the triggering object (CVMR_passes), untimely responding (CVMR_falstart). There was one more type of errors that was specific to CVMR. It was a false reaction to the triggering stimulus of the wrong color, i.e., the responding to the red flash rather than to the green one (CVMR_false_reaction).
  Normally, CVMR takes more time than SVMR; the difference in their length is called *decision-making time (DMT)* (see Equation 1). It reflects the time cost of *response selection* (see Figure 1). The process affiliates the cognitive subdomains of task switching and inhibitory control, i.e., the examinee inhibits prepotent responses and shifts between tasks.
  The number of incorrect responses is expected to be higher in CVMR than in SVMR. For both SVMR and CVMR, one can calculate the percentage of trials that went wrong in the overall number of trials. To compare performance in the tests, one may use a derivative variable called inverse efficiency score (IES) (see Equation 2).
3. *Reaction to a moving object (RMO)* allows us to estimate a balance or predominance of either excitation or inhibition in the central nervous system. At the time of the test, a circle, a starting point and a finishing line appear on the screen. The circle is quickly filled at a constant radial pace with a color, from some starting point to the finishing line in a clockwise direction as shown on Figure 2. The examinee is asked to respond by pressing the button the moment the targeted moving object crosses the finishing line. In a set of subsequent attempts, the response time delays are documented as positive values, whereas the premature responses are documented as negative values.
  There are a total number of over 30 trials (RMO_trialsNo). The system calculated the mean value (RMO_mean) of positive (the time delays) and negative values (the premature responses). If the RMO_mean value is positive, it reveals the predominance of inhibition over excitation. When negative, RMO_mean indicates the predominance of excitation over inhibition.
  The application sums up the number of the delayed responses (RMO_delays), the false starts (RMO_falstart) and the total number of responses that were accurate in time (RMO_acc). Also, the tester records the time length of the responses (RMO_delaysTotalTime, RMO_falstartTotalTime, and RMO_positiveSum).
4. *Attention study technique*: To test attention, identical triggering stimuli are presented subsequently in different locations on a computer screen (see Figure 3A). The mean response time (AST_mean) reflects the level of attention to the visual objects, stability, concentration of attention, speed of information processing, and work efficiency.
5. *Interference resilience technique*: In contrast to the previous task, this technique includes additional interfering objects (e.g., circles of different color and size) overlapping each other and the targeted stimuli, which requires additional time for the participant to notice the triggering signal and respond (see Figures 3B,C). The system calculates the average response time (IRT_mean).
6. We used *wrist dynamometry* to measure the *maximum muscular strength* of the right (WDR_MMS) and left hand (WDL_MMS). *Asymmetry coefficient* (AC) is calculated as the ratio of the maximum muscular strength of the wrists (see Formula 4).

(1) $$\begin{array}{r} DMT=CVMR\text{\_}mean-SVMR\text{\_}mean \end{array}$$

(2) $$IES=\frac{Mean\;value\;of\;the\;reaction\;time}{1-mistakes,\%}$$

(3) $$\begin{array}{r} TRVI=IRT\text{\_}mean-AST\text{\_}mean \end{array}$$

(4) $$\begin{array}{r} AC=\frac{WDR\text{\_}MMS}{WDL\text{\_}MMS} \end{array}$$

### 3.2. Characteristics of the Study Participants

We analyzed the data from a publicly available dataset named after the title of the project Psychophysiological Outcomes of Brain Atrophy (POBA). The dataset contains about 100 features reflecting the overall psychophysiological status of 231 people of different age (4–83 years; 134 women, 97 men). Written patient consent or parental consent with assent from minors for testing and scanning was obtained in each case. The examinees were scanned to exclude brain pathology. Some of them suffered from periodic headaches. The others started their professional sports career. Not to impact the study outcomes, the examinees were not paid for participation or being tested.

*The inclusion criterion* was literacy, i.e., only those adults who indicated that they did at least professional courses after finishing general education took part in the study.

*The exclusion criteria* were as follows: organic brain pathology, mental disorders, and recent or past injury to the head. All the participants were examined by a qualified neurologist. They were found healthy based on the clinical examination and the negative results of MRI which was conducted to rule out any underlying pathology. In this way, we followed the recent practice parameters of the American Academy of Neurology that recommend neuroimaging (e.g., MRI) to detect neurodegenerative conditions in early stages (Fuller et al., 2019). Supplementary Table 1 shows the distribution of the subjects across age and sex groups. The dataset is provided on demand (see section 9). Additional details on the study design and description of all the features are available in our recent studies (Statsenko et al., 2020, 2021a).

### 3.3. Methodology of the Study

**Figure 4:**
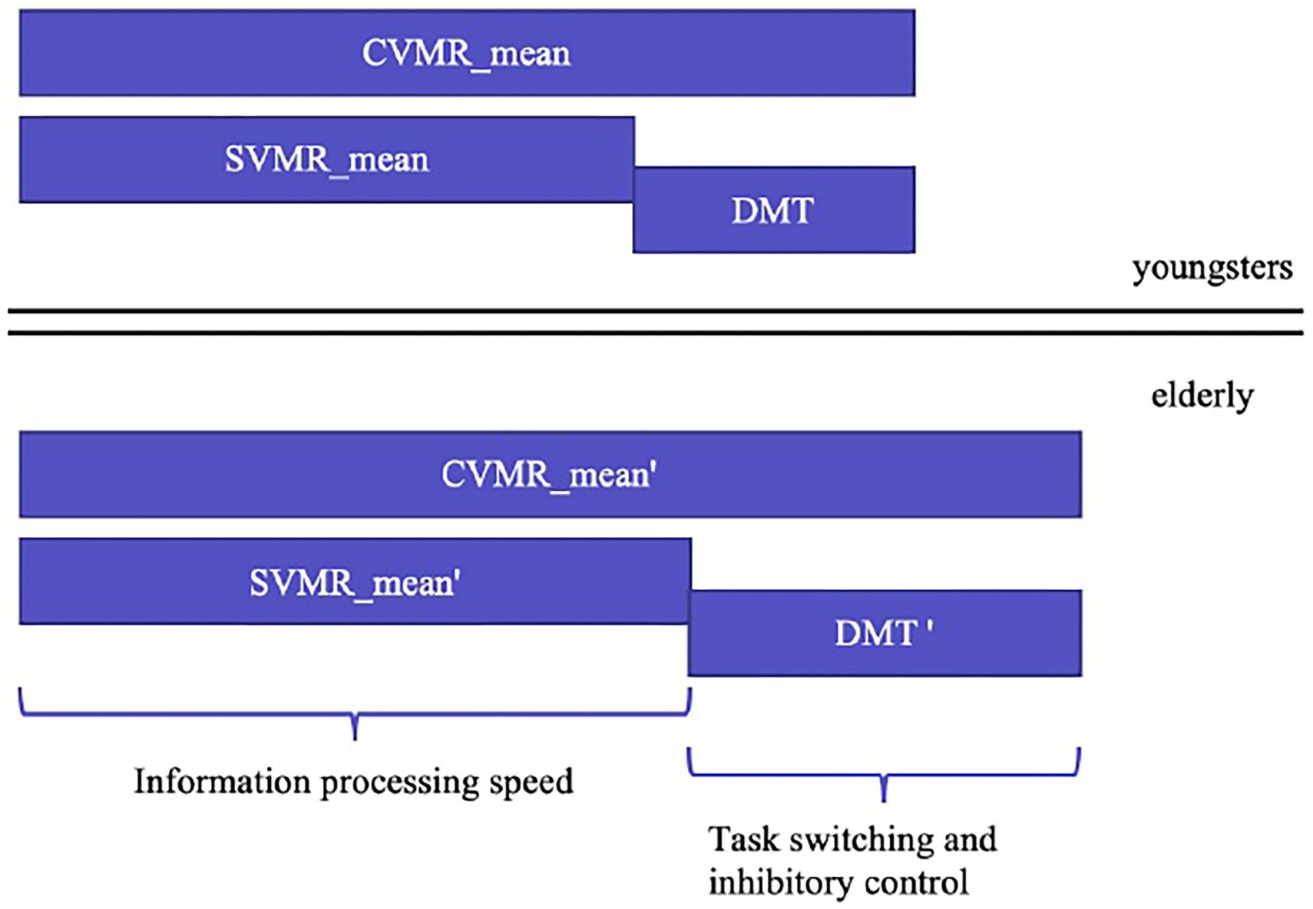
Age-related changes in simple and complex visual-motor tasks estimates and the cognitive functions they reflect.

*To find the appropriate solution for the first objective*, we analyzed the structure of the complex visual-motor reaction (CVMR), which is also commonly called the choice reaction. Similar to the simple visual-motor reaction, the choice reaction includes such components as sensory acquisition (visual perception) and motor responding. Additionally, CVMR encompasses the decision-making component required to process an inhibitory condition present in the task (see Figure 4). This processing causes a time delay (DMT). In our recent study, we analyzed the age-related variability of DMT. In this study, we want to concentrate on the ratio between DMT and SVMR time, because they reflect different cognitive functions: the switching and inhibitory control estimate vs. the information processing speed estimate.

We also found the analysis of RMO_mean variable relevant to this study. In the RMO task, the examinee is supposed to respond subsequently to a set of expected events accurately at the anticipated moments when the events occur. To perform the test, the individual employs such cognitive domains as attention, inhibitory control, and task switching. Thus, the dependent variable of the *reaction to a moving object test* (RMO_mean) reflects the cooperative involvement of different cognitive domains.

*The second objective* was to find the optimal number *k* of distinct nonoverlapping subgroups with regard to age. For this, we used a heuristic approach based on the elbow method. We utilized the K-means algorithm and evaluated the distortion score or the sum of squared distances from each point to its assigned centroid as a performance metric of each chosen *k*. The optimal cutoff *k* value corresponded to the minimal distortion score. When the optimal *k* was set, we utilized the K-means clustering method to assess the separability measure with regard to the age group by finding a centroid for each cluster and counting the number of relevant and irrelevant data points in the groups.

*To address the third objective*, we analyzed the charts that describe age-related changes of the aforementioned indices and dependent variables. Using the bootstrap method with regard to age, we built the linear regression model fit and 95% CI for the regression estimate. Then we analyzed the progression or dynamics of the proposed indices. To compare lifelong changes of the variables in different age-groups, we used descriptive statistics and the Kruskal-Wallis test (Habuza et al., 2021c). To figure out whether there is statistically significant differences between the data for two sexes, we resorted to statistical hypothesis tests, specifically *t*-test.

*The fourth objective* was multifold. We hypothesized that the newly developed indices may reflect age-related changes of the psychophysiological status of the examinees. To look for the possible associations of the age, indices, and other psychophysiological tests, we fed these data to conventional ML models, trained with a 10 fold cross-validation technique, and compared their performance.

*In the first part of the fourth objective*, we used ML classification algorithms to predict the age group from an individual performance in PTs. The threshold value was 40 years of age. Our recent study justifies this age to be a cut-off value for cognitive decline that can be identified from test performance (Statsenko et al., 2021a). In this study, we utilized *classification models*. For this, we added a single newly proposed index to the list of predictors used in the recent study (see the left column in Supplementary Table 2). We employed conventional ML classification models with default architecture from Python scikit-learn v. 0.24.2 library. The classifiers we used are listed in comments to **Table 5**. We trained all the models in the stratified k-fold cross-validation technique until convergence as per scikit-learn default settings. The results predicted in each fold were merged and then averaged to report the final accuracy of the models. By comparing the model performance metrics, we tested the informative input of the indices to the prediction of the age. To evaluate the performance of the predictive models, we generated a receiver operating characteristic (ROC) curve averaged over 10 times. We also calculated the mean sensitivity, specificity, accuracy (ACC), and area under the curve (AUC) values. These performance measures were suitable as the dataset was balanced across the age attribute. Then, we averaged the accuracy of the models by calculating *Mean* ± *SD* for each index used as a predictor (see the left columns of **Table 5**). Finally, with the Kruskal-Wallis test we tested whether the variance of the accuracy differs significantly (*p* > 0.05) for an index compared to other indices.

*Working on the second part of objective four*, we figured out whether the proposed indices provide a summary of the findings obtained while testing individuals. To address the task, we used ML regression models forecasting values of the newly proposed indices. As predictors, we utilized the features comprising POBA dataset with an exception of those from which the values of the indices can be calculated. Specifically, to predict the values of ISD and ISDA indices we used the variables listed in the middle column of Supplementary Table 2. To forecast the value of ISCA index, we used the same list of predictors with an exception of “SVMR_IES” as the prognosis can be calculated from this feature. To evaluate the quality of regressor outcome, we employed mean absolute error (MAE), root mean squared error (RMSE), and a proportion of MAE to the range of values (see the right columns of **Table 5**).

### 3.4. Hardware and Software Used

All the experiments were conducted with the Linux Ubuntu 18.04 workstation with 24 CPU cores and two NVIDIA GeForce GTX 1080 Ti GPU with 11 GB GDDR5X memory each using programming language Python, and its libraries for data processing, ML, and data visualization, such as scikit-learn, NumPy, Pandas, Matplotlib, Seaborn, and Yellowbrick.

## 4. Results

### 4.1. Estimates of the Dis/Proportional Changes in Cognitive Domains

Based on the analysis described in section 3.3, we came up with the *index of simple reaction time to decision-making time (ISD)*. It provided us with the ratio of processing speed to decision-making time (see Equation 5). Both the time estimates were vulnerable to age-related neurocognitive slowing; however, there are no clear data supporting the assumption that the pace of decline is equal in diverse cognitive domains. Therefore, the derivative variable may serve as a marker of their disproportional decline.


(5) $$\begin{array}{r} ISD=\frac{SVMR\text{\_}mean}{DMT} \end{array}$$

ISD index considers two indicators that constitute the reaction time of the visual-motor task with the switching condition in it. The weak point is that it does not take the performance accuracy into consideration. For this reason, we proposed an additional derivate variable, which was the *index of simple reaction time to decision-making time with the accuracy performance (ISDA)*. If compared with the previous one, the index includes the percentage of correct responses in the denominator (see Equation 6).


(6) $$\begin{array}{r} ISDA=\frac{SVMR\text{\_}mean}{DMT\times \left(1-CVMR\text{\_}mistakes,\%\right)} \end{array}$$

Another way to combine the speed and accuracy estimates of SVMR and CVMR is to calculate the IES score for each of the tests separately and then find the ratio between them. This solution leads us to the *index of performance in simple and complex visual-motor reaction with account for accuracy (ISCA)* calculated as it is seen in Equation 7.


(7) $$\begin{array}{r} ISCA=\frac{IES_{SVMR}}{IES_{CVMR}}=\frac{SVMR\text{\_}mean\times \left(1-CVMR\text{\_}mistakes,\%\right)}{CVMR\text{\_}mean\times \left(1-SVMR\text{\_}mistakes,\%\right)} \end{array}$$

In this research, we did not intend to test all the possible ratios of the performance metrics in different cognitive tasks. Our goal was to show the utility of the approach when cognitive testing covers several domains and estimates their interrelated divergent changes.

### 4.2. Identification of the Optimal Number of Age Cohorts

In the cluster analysis, we used two variables (age and a proposed index) and employed K-means method to segregate the data points between groups. We utilized the unsupervised learning clustering K-means method to find the optimal number of homogeneous groups of data points. With the elbow method, we found the most appropriate number of clusters. It was justified by the separability measure based on a distortion score. The score is the sum of squared distances from each point to its assigned centroid. All centroids are obtained iteratively by minimizing the intracluster proximity while maximizing the distance between clusters. We applied the elbow method to two-dimensional data points composed of the age of the subjects and corresponding index values, where the *index* was set to ISD, ISDA, or ISCA.

**Figure 5:**
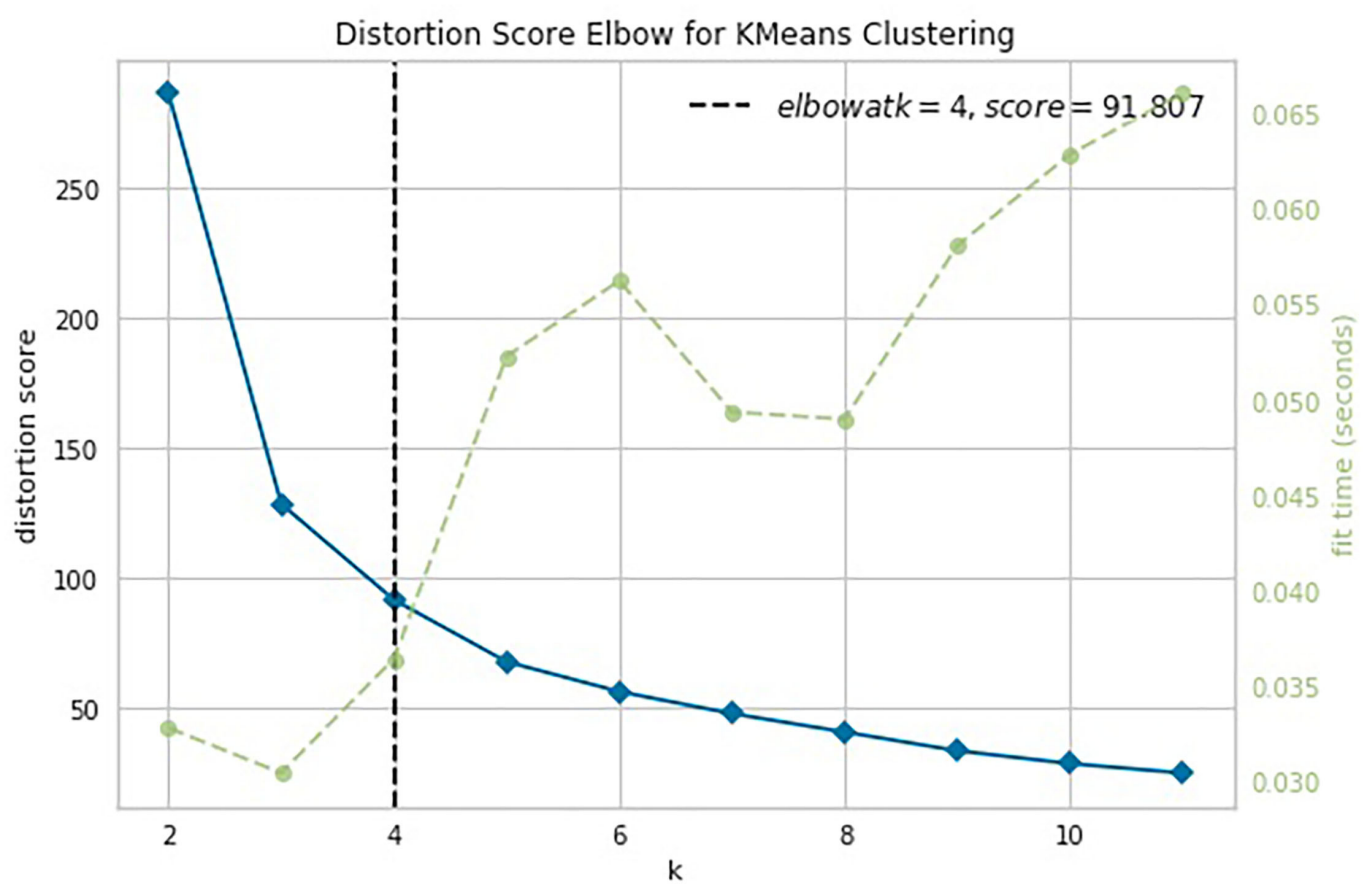
Selection of the optimal number of clusters by using the Elbow method with the knee point detection algorithm for ISCA index.

The knee point detection algorithm (Satopaa et al., 2011) returns the optimal value of clusters equal to four for each proposed indices. In Figure 5, built for the data points (*age, ISCA*), the optimal choice is annotated with a black dashed line. The blue line on the graph is plotted from the values of distortion scores with regard to the number of clusters, whereas the green dashed line displays the amount of time needed to train the clustering model per *k*.

The participants in our sample were not uniformly distributed over age; however, with the proper choice of bin width, the age histogram can become very close to the uniform one. Another concern is related to the number of subjects who were over the age of 75 years old, a relatively small subgroup, in contrast to the number of participants who were under 15. To get a relatively equal number of participants in each group, we decided to count the 20 years long intervals from birth rather than from the age of the youngest examinee.

We assessed the performance of the clustering method by looking at the values of the aforementioned indices as predictors of age-groups. We plotted the points returned by the clustering method with their centroids and thoroughly analyzed their age values. The centroids were almost uniformly scattered on the age axis with the step of approximately 20 years (see Table 2). The centroid coordinates were calculated as the average value of all the points in the corresponding cluster; therefore, we used the obtained age granulation to compose our groups. In such a manner, we segregated four groups in our sample: Adolescent ∈ [0, 20), Young adults ∈ [20, 40), Midlife adults ∈ [40, 60), and Older adults ≥60. With this division in Table 2, we presented the number of points correctly identified by the clustering method (performance column) vs. misclassified points (misclassified column). The best performance was obtained on the ISCA. Only five cases of Midlife adults were misclassified as Young adults. So, the ISCA index reflects the age-related psycho-physiological changes reliably.

**Table 2:** Clusterization of the sample into age cohorts with regard to the variables describing the proportion between cognitive functional activities and cooperative involvement of cognitive domains in tasks.

**Group**	**Capacity** **(females:males)**	**ISD Index**			**ISDA Index**			**ISCA Index**		
		**Centroid**	**Performance**	**Misclassified**	**Centroid**	**Performance**	**Misclassified**	**Centroid**	**Performance**	**Misclassified**
Adolescents	48 (19:29)	11.853	48	0	12.047	48	0	11.661	48	0
Young adults	64 (36:28)	31.302	62	2	31.600	60	4	30.075	64	0
Midlife adults	64 (39:25)	53.089	57	7	53.529	56	8	49.908	59	5
Older adults	55 (40:15)	70.647	46	9	71.018	46	9	68.274	55	0

### 4.3. Proportionality of Age-Related Changes in the Cognitive Domains

**Figure 6:**
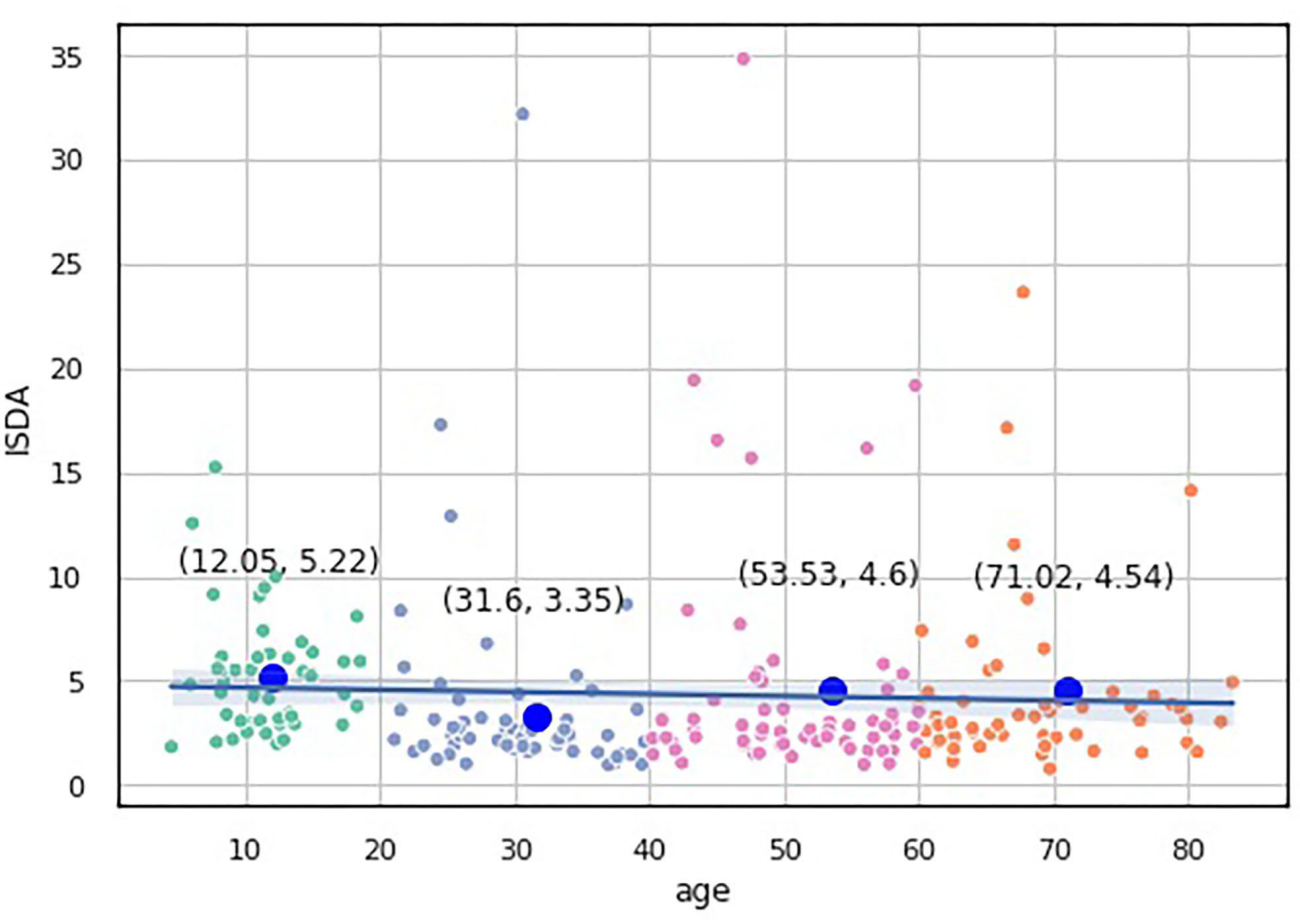
Over-the-age distribution of ISDA values.

**Figure 7:**
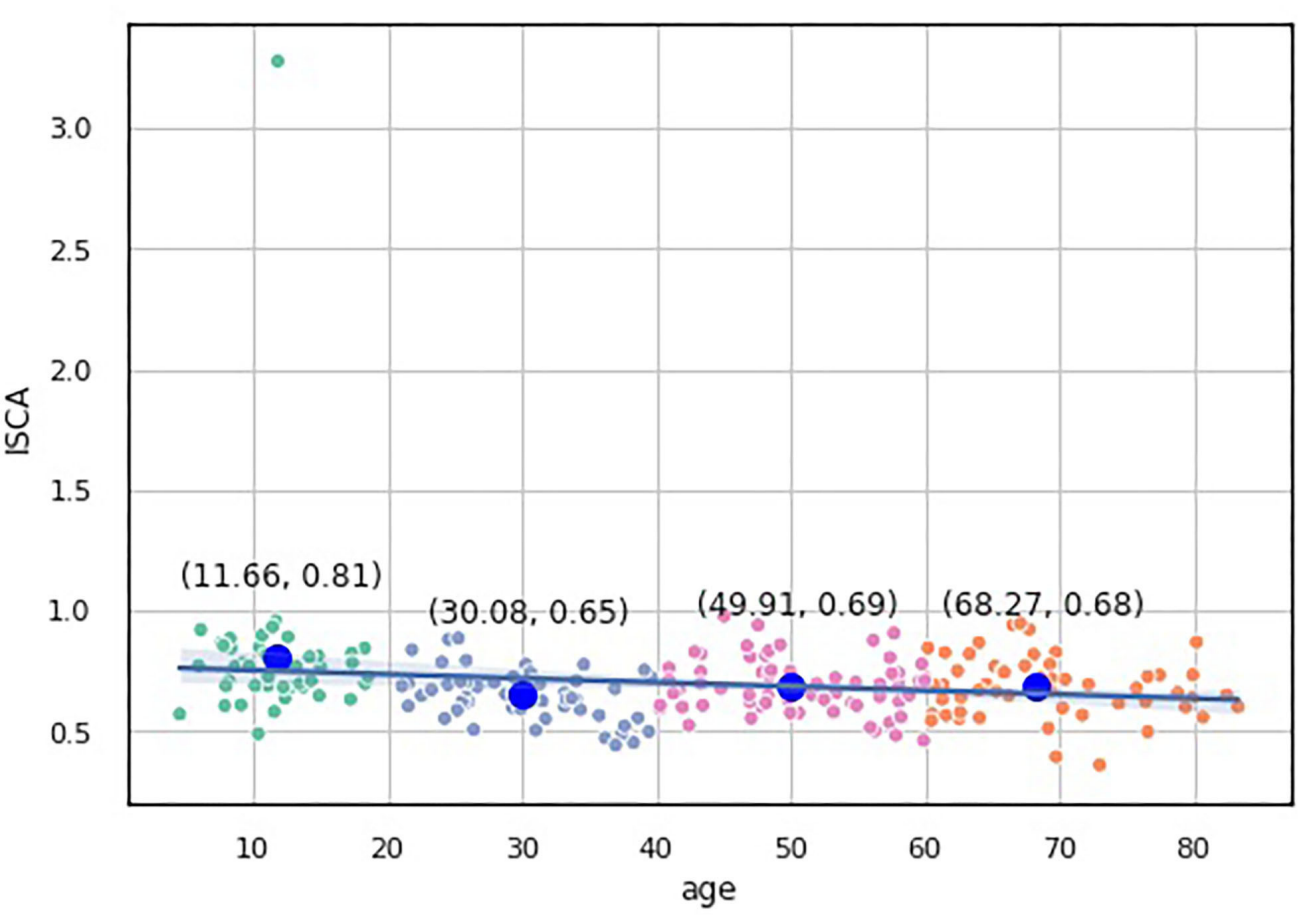
Over-the-age distribution of ISCA values.

We built a pairwise distribution of each proposed index with age (see Figures 6, 7). The linear horizontal trendlines with a 95% CI for the linear regression model estimates represent tendencies toward sustaining a balance between cognitive functions corresponding to diverse interrelated domains.

To compare the distribution of the indices over the age-groups, we applied statistical significance tests. Because none of our indices data underwent Gaussian distribution according to the Shapiro-Wilk test for normality (*p* < 0.05), we utilized nonparametric statistics. First, we checked the hypothesis that the population medians of all the groups were equal to the Kruskal-Wallis test, which showed significant changes in the distribution of the four groups (*p* < 0.05).

To study which groups differed in their medians, we ran *post hoc* Dunn test with the step-down method. We used Bonferroni adjustments (Holm's step-down procedure) to control the family-wise error rate. Regarding the indices values, only the median of the Adolescents group differed from those of the other three groups in the indices values. The three remaining groups shared a similar distribution pattern (*p* > 0.05). After a period of neurodevelopmental changes and maturation, the indices preserve almost constant values with a slight trend toward functional decline.

**Figure 8:**
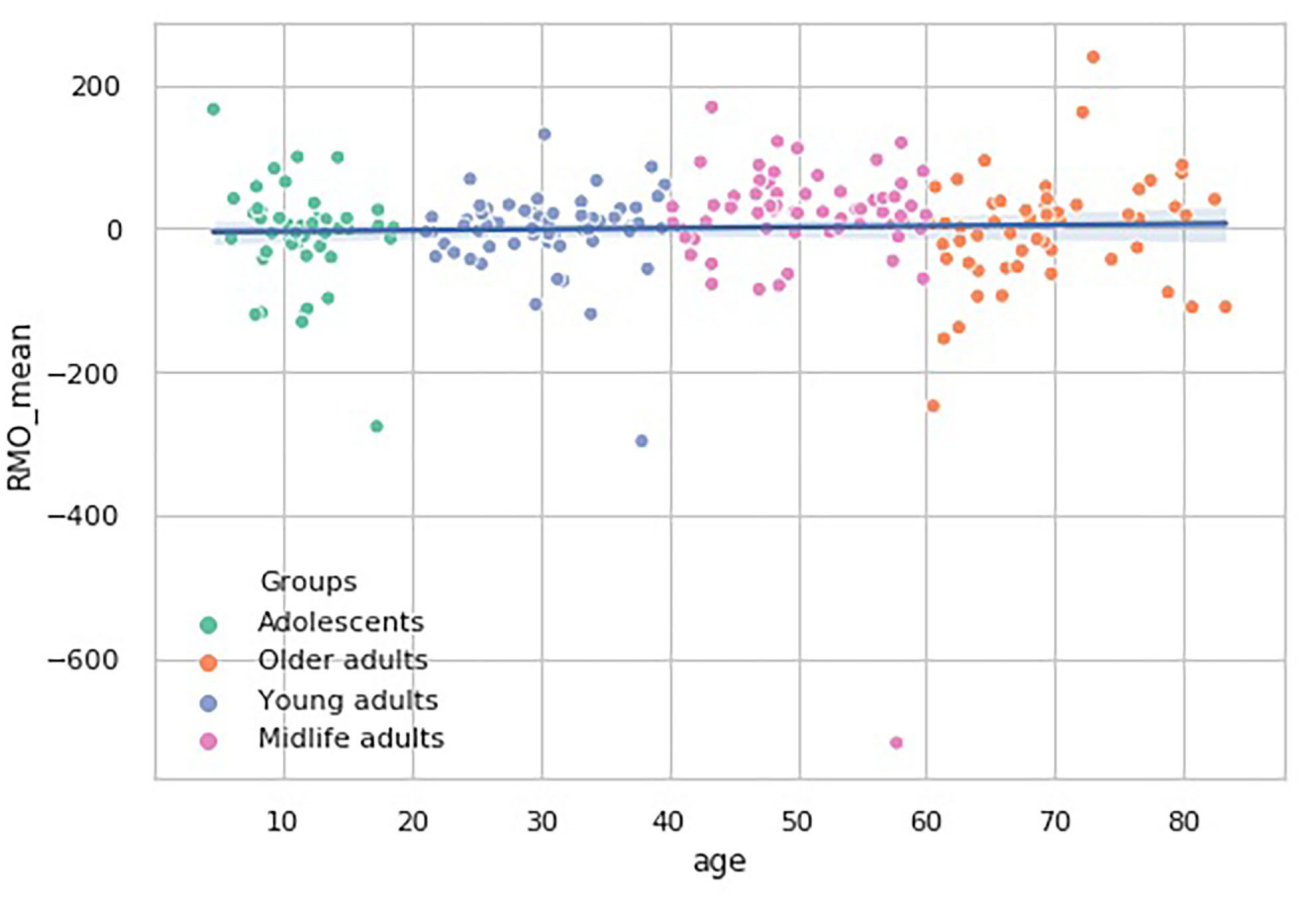
Over-the-age distribution of time estimate values in RMO test.

In the RMO test, however, there is another tendency. The RMO_mean values pairwise comparisons show that the Midlife adults group median significantly differs from the remaining three age subsamples (*p* < 0.01). No general trend in age-related changes of this dependent variable is observed (see Figure 8).

Finally, we investigated the variances of the proposed indices and selected psychophysiological attribute values for different groups. Levene's test reveals no significant changes (*p* > 0.05) in the variances among age-groups for the aforementioned values. Homoscedasticity also supports our assumption about stable linear dependency between the observed features and age.

#### 4.3.1. Age-Related Trends in Cognitive Subdomains and Proportionality of Their Changes

The information on statistically significant differences between the age-groups in the performance in PTs is presented in Table 3.

**Table 3:** Comparison of results of PTs.

	**Total**	**Adolescents**	**Young adults**	**Midlife adults**	**Older adults**	**p_**1**_−_**4**_**
	***n* = 231**	**n_**1**_ = 48(20.78%)**	**n_**2**_ = 64(27.71%)**	**n_**3**_ = 64(27.71%)**	**n_**4**_ = 55(23.81%)**	
**Performance in psychophysiological tests**						
SVMR_mean	260.51 [219.63–285.83]	282.03 ± 70.91^*^	221.03 ± 28.92^*^	259.76 ± 55.48	288.52 ± 53.75^*^	1.61005e-14
SVMR_variance	69.88 [41.09–80.82]	89.01 ± 73.36	49.41 ± 22.39^*^	67.69 ± 36.54	79.54 ± 42.92^*^	7.05157e-06
SVMR_mistakes	1.32 [0.0–2.0]	2.69 ± 3.83^*^	0.83 ± 1.32^*^	0.62 ± 1.11^*^	1.49 ± 1.54^*^	2.8462e-06
SVMR_IES	280.06 [224.94–304.73]	339.43 ± 236.3^*^	227.9 ± 32.9^*^	265.77 ± 59.02	305.56 ± 64.35^*^	7.00503e-15
CVMR_mean	360.77 [307.45–395.57]	360.8 ± 107.74	324.89 ± 56.55^*^	362.64 ± 65.15	400.32 ± 71.9^*^	9.41694e-08
CVMR_variance	108.91 [70.7-118.64]	121.55 ± 94.58	91.82 ± 80.43^*^	92.65 ± 30.46	136.69 ± 74.86^*^	2.0683e-07
CVMR_mistakes	2.87 [1.0–4.0]	3.65 ± 2.45^*^	2.58 ± 2.81^*^	2.14 ± 1.75^*^	3.4 ± 2.26^*^	0.000253234
CVMR_IES	402.91 [336.52–448.65]	416.17 ± 143.57	359.93 ± 81.36^*^	390.66 ± 66.29	455.62 ± 95.44^*^	5.88309e-09
DMT	100.26 [63.6-122.43]	78.76 ± 52.97^*^	103.86 ± 48.64	102.88 ± 51.65	111.79 ± 57.81	0.00056484
RMO_mean	0.32 [-18.5–31.35]	−8.99 ± 69.28	−2.14 ± 54.25	12.73 ± 104.22^*^	−3.12 ± 75.59	0.00646979
RMO_variance	167.86 [84.7-224.35]	168.85 ± 103.5	111.84 ± 67.33^*^	158.75 ± 93.83	242.81 ± 105.18^*^	5.6846e-12
RMO_errors	20.95 [18.0–24.0]	19.96 ± 5.22	18.14 ± 4.14^*^	22.22 ± 3.82^*^	23.62 ± 3.34^*^	5.24218e-11
**Proportionality of changes in cognitive subdomains**						
ISD	3.82 [1.97–4.13]	4.53 ± 2.29^*^	3.02 ± 2.98^*^	4.14 ± 4.9	3.76 ± 3.59	5.53179e-06
ISDA	4.35 [2.15-4.87]	5.22 ± 2.75^*^	3.57 ± 4.48^*^	4.55 ± 5.65	4.26 ± 3.99	8.10003e-07
ISCA	0.7 [0.61–0.77]	0.81 ± 0.37^*^	0.65 ± 0.1^*^	0.68 ± 0.11	0.68 ± 0.12	1.82596e-05

*Data for different age-groups are expressed as Mean ± SD*.

* *If the distribution of performance metrics differs significantly (p < 0.05) compared to other cases taken together, its Median ± SD is marked with an asterisk*.

#### 4.3.2. Sex-Related Traits in Cognitive Subdomains and Proportionality of Their Changes

Table 4 shows the descriptive statistics on the tests performance in both sexes.

**Table 4:** Performance in PTs with regard to sex and age.

	**Total**			**Adolescents**			**Young adults**			**Midlife adults**			**Older adults**		
	**Female**	**Male**	**p_**1**__**−**__**2**_**	**Female**	**Male**	**p_**3**__**−**__**4**_**	**Female**	**Male**	**p_**5**__**−**__**6**_**	**Female**	**Male**	**p_**7**__**−**__**8**_**	**Female**	**Male**	**p_**9**__**−**__**1**__**0**_**
	***n_**1**_* = 134**	***n_**2**_* = 97**		***n_**3**_* = 19**	***n_**4**_* = 29**		***n_**5**_* = 36**	***n_**6**_* = 28**		***n_**7**_* = 39**	***n_**8**_* = 25**		***n_**9**_* = 40**	***n_**1**__**0**_* = 15**	
Proportion	58.01%	41.99%		39.58%	60.42%		56.25%	43.75%		60.94%	39.06%		72.73%	27.27%	
Age	44.89 ± 20.08	36.18 ± 21.98	**6.8e-4**	11.64 ± 3.41	11.68 ± 3.35	0.368	30.82 ± 4.72	29.12 ± 5.38	0.105	50.56 ± 5.67	50.87 ± 6.68	0.371	67.84 ± 5.7	72.26 ± 7.72	**0.037**
**Performance in psychophysiological tests**															
SVMR_mean	265.33 ± 57.56	253.84 ± 61.31	**0.015**	290.9 ± 82.47	276.23 ± 61.5	0.433	224.4 ± 25.96	216.69 ± 31.8	**0.045**	269.65 ± 50.34	244.34 ± 59.48	**0.004**	285.83 ± 50.36	295.71 ± 61.31	0.342
SVMR_variance	70.82 ± 47.22	68.57 ± 48.48	0.356	100.08 ± 82.66	81.75 ± 65.55	0.392	49.26 ± 21.14	49.6 ± 23.9	0.386	71.5 ± 36.82	61.76 ± 35.31	0.145	75.68 ± 40.62	89.85 ± 47.01	0.091
SVMR_mistakes	1.13 ± 1.57	1.58 ± 2.91	0.158	2.32 ± 2.23	2.93 ± 4.57	0.470	1.06 ± 1.51	0.54 ± 0.94	0.084	0.49 ± 1.13	0.84 ± 1.05	**0.031**	1.25 ± 1.24	2.13 ± 2.0	0.099
SVMR_IES	278.03 ± 72.12	282.86 ± 172.73	**0.045**	321.21 ± 112.6	351.37 ± 289.41	0.466	233.56 ± 33.02	220.62 ± 31.27	**0.020**	275.16 ± 57.34	251.12 ± 58.62	**0.018**	300.34 ± 63.59	319.48 ± 64.27	0.130
CVMR_mean	369.13 ± 81.17	349.22 ± 77.4	**0.026**	375.09 ± 142.16	351.43 ± 75.82	0.450	331.23 ± 47.76	316.73 ± 65.26	0.123	371.52 ± 60.98	348.79 ± 68.91	**0.046**	398.07 ± 68.13	406.3 ± 80.78	0.407
CVMR_variance	110.19 ± 79.9	107.14 ± 67.4	0.380	102.73 ± 77.28	133.87 ± 102.52	*0.052*	96.51 ± 102.58	85.78 ± 34.53	0.226	98.4 ± 33.17	83.68 ± 22.95	0.062	137.54 ± 83.25	134.41 ± 45.39	0.194
CVMR_mistakes	2.52 ± 2.49	3.36 ± 2.23	**3.3e-4**	3.0 ± 2.66	4.07 ± 2.2	*0.057*	2.67 ± 3.26	2.46 ± 2.1	0.351	1.74 ± 1.63	2.76 ± 1.75	**0.009**	2.92 ± 2.09	4.67 ± 2.21	**0.004**
CVMR_IES	408.37 ± 110.89	395.36 ± 92.34	0.248	427.72 ± 195.46	408.6 ± 94.56	0.205	371.76 ± 91.25	344.72 ± 63.34	0.167	395.57 ± 67.73	383.0 ± 63.23	0.201	444.63 ± 90.66	484.91 ± 101.49	*0.059*
DMT	103.8 ± 58.5	95.38 ± 46.4	0.283	84.19 ± 71.55	75.2 ± 35.47	0.483	106.84 ± 44.24	100.03 ± 53.53	0.269	101.87 ± 56.28	104.45 ± 43.4	0.216	112.25 ± 62.59	110.59 ± 42.51	0.392
RMO_mean	−2.72 ± 91.16	4.53 ± 58.05	0.415	−11.31 ± 90.65	−7.48 ± 50.55	0.288	−1.96 ± 63.88	−2.37 ± 38.5	0.262	−5.11 ± 124.22	40.56 ± 49.66	**0.034**	2.99 ± 71.71	−19.44 ± 82.9	0.288
RMO_variance	183.45 ± 107.07	146.33 ± 95.13	**0.001**	198.95 ± 123.68	149.12 ± 82.02	0.115	114.94 ± 70.91	107.85 ± 62.19	0.325	182.65 ± 96.42	121.47 ± 75.85	**5.5e-4**	238.53 ± 100.88	254.22 ± 115.11	0.356
RMO_errors	21.96 ± 4.25	19.57 ± 4.83	**4.9e-5**	20.95 ± 5.71	19.31 ± 4.77	0.097	18.72 ± 3.91	17.39 ± 4.3	0.116	23.36 ± 2.99	20.44 ± 4.27	**0.003**	23.98 ± 2.55	22.67 ± 4.71	0.191
**Proportionality of changes in cognitive subdomains**															
ISD	3.92 ± 4.13	3.68 ± 2.95	0.360	4.59 ± 2.35	4.5 ± 2.24	0.466	2.75 ± 2.75	3.36 ± 3.23	0.292	4.66 ± 5.53	3.34 ± 3.57	0.130	3.93 ± 4.01	3.29 ± 2.03	0.399
ISDA	4.42 ± 5.09	4.24 ± 3.47	0.214	5.14 ± 2.69	5.27 ± 2.79	0.376	3.42 ± 5.04	3.76 ± 3.63	0.340	5.05 ± 6.36	3.75 ± 4.21	0.186	4.37 ± 4.43	3.95 ± 2.41	0.251
ISCA	0.69 ± 0.12	0.71 ± 0.28	0.141	0.77 ± 0.1	0.83 ± 0.47	0.212	0.64 ± 0.09	0.65 ± 0.1	0.386	0.7 ± 0.12	0.65 ± 0.08	**0.036**	0.69 ± 0.13	0.67 ± 0.11	0.184

*Data for the cohorts are expressed as Mean ± SD*.

*The significant differences between the sexes are marked in bold. The differences close to significant are marked in italic*.

It is interesting to note that performance in the majority of the PTs does not differ significantly (*p* > 0.05) between women and men with an exception of the group of Midlife adults.

In the age interval [40, 60) years, women seem to spend more time on the task on average to compensate for a better accuracy in both SVMR and CVMR tests. Women were also more accurate in responding to RMO test stimuli (RMO_mean was −5.11 ± 124.22 vs. 40.56 ± 49.66; *p* = 0.034). However, the variance of the reaction time and the number of errors was significantly lower in men (182.65 ± 96.42 vs. 121.47 ± 75.85; 23.36 ± 2.99 vs. 20.44 ± 4.27).

In the same age group, ISCA index was significantly higher in women compared to men (0.7 ± 0.12 vs. 0.65 ± 0.08; *p* = 0.036). However, this difference does not seem to be considerable as the mean values for both sexes stay close within the interquartile range. No other essential differences were found between the values of the indices for men and women of any age group.

### 4.4. Informative Value of the Indices Developed

Our idea was to inspect possible relationship of ISD, ISDA, and ISCA both with the general psychophysiological status of the examinee and the age of the individual. For getting an insight into this, we resorted to machine learning approach.

#### 4.4.1. Associations of the Newly Proposed Indices With Age

We tried to estimate the potential of the variables derived from the test results to reflect the entire psychophysiological status of the individual. As the individual psychophysiological status undergoes age-related changes, one may expect that the derivative indices should also reflect this process. In this study, we trained classification models to predict the age group of the examinee, i.e., whether it is below or above 40 years of age. The utility of the cut-off level has been already justified in our recent studies (Statsenko et al., 2021a). By feeding the models with the data of the novel indices, we analyzed the information value of the latter for such a prediction. The performance metrics of the models are presented on the left side of Table 5.

**Table 5:** Performance of the classification and regression models.

**Classification by age group** **below and above 40 years**					**Regression models**			
**Predictor** **used**	***Sens*.**	***Spec*.**	**ROCAUC**	**ACC**	**Forecasted** **variable**	**MAE**	**RMSE**	$\frac{MAE}{range},\%$
ISD	0.7 ± 0.056	0.73 ± 0.03	0.78 ± 0.04	0.715 ± 0.29	ISD	2.15 ± 0.14	3.56 ± 0.31	7.62 ± 0.5
ISDA	0.72 ± 0.06	0.73 ± 0.04	0.8 ± 0.03	0.727 ± 0.28	ISDA	2.58 ± 0.19	4.34 ± 0.44	7.56 ± 0.55
ISCA	0.73 ± 0.04	0.73 ± 0.03	0.8 ± 0.02	0.73 ± 0.024	ISCA	0.102 ± 0.004^*^	0.18 ± 0.013^*^	3.49 ± 0.14^*^

* *The performance is expressed as Mean ± SD values among the following classifiers and regressors: Gradient Boosting, Gaussian NB/AdaBoost, Ridge/Lasso, SVM linear/LR, Random Forest and SVM (non-linear)*.

*If the distribution of metrics differs significantly (p < 0.05) for an index compared to other ones, its Mean ± SD is marked with an asterisk*.

#### 4.4.2. Predictive Potential of PTs to Identify the Values of Proposed Indices

We tried to estimate whether the variables derived from the test results could reflect the entire psychophysiological status of the individual. As the battery of PTs describes the individual psychophysiological status, we trained a regression model to predict the values of the proposed indices. The performance metrics are presented on the right side of Table 5.

**Figure 9:**
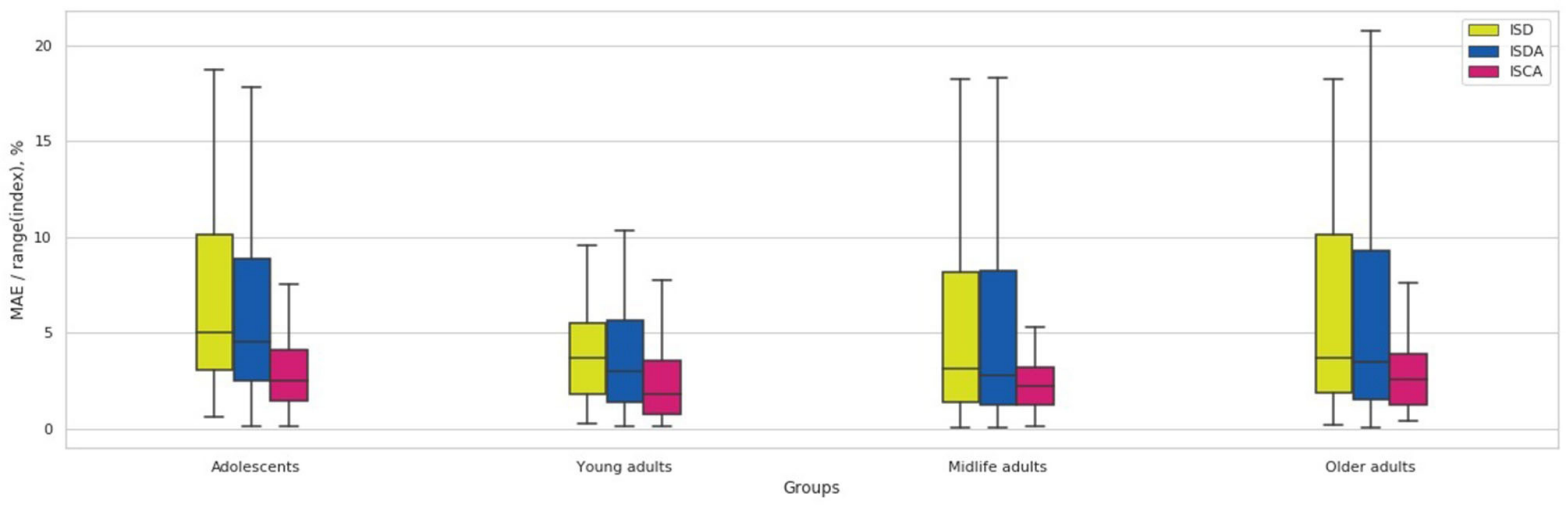
Over-the-age-groups distribution of MAE/range(index) values for Random Forest regression model.

The performance metrics of the regression model such as mean absolute error (MAE), root mean squared error (RMSE), and the coefficient of determination (R2) are presented on the right side of Table 5. The notched boxplot in Figure 9 reveals the accuracy of the prediction in terms of the proportion of MAE to the range of the index in different age-groups. Its distribution over the age-groups justifies the hypothesis that ISCA reflects the psychophysiological status more reliably than ISD or ISDA do. From Table 5, the MAE-to-range of values proportion is significantly smaller in ISCA (3.49 ± 0.14% vs. 7.62 ± 0.5% in ISD and 7.57 ± 0.55% in ISDA; *p* < 0.05). In Figure 9, both the interquartile range and the CI are significantly smaller in any age group for ISCA compared to the other indices.

## 5. Discussion

In this study on aging, we resorted to psychophysiological tests. This goes in line with the recent publications (McKinney et al., 2019; Rech et al., 2020) that show that sensorimotor activity studies are a promising area of research in gerontology. The tests we used cover two related but not identical spheres: individual cognition and emotions.

*Individual cognition* is a complex functional system in which some blocks account for the basic implementation of sensorimotor activity, while other blocks coordinate an interaction of the sensory and motor components. The components of sensorimotor response are closely interrelated with the high-level mental functions (Mitchell et al., 2019) that underlie the efficiency of the activity and imply for the performance of the sensorimotor test. This makes it possible to use the tests for the functional assessment of the central nervous system in aging (Cassady et al., 2019, 2020) and professional activities (Li et al., 2019; Boichuk et al., 2020; Myroshnychenho et al., 2020).

*The personal emotional status* assessment is also possible with PTs. Testing sensorimotor activity aims at overcoming the limitations of the formal systematic assessment used in classical psychology (Romero et al., 2019; Baksheva et al., 2020).

The battery of tests used in the study is strongly associated with other cognitive metrics and appears to be a more reliable predictor of important social and health outcomes than other tests (Deary and Der, 2005; Der and Deary, 2006). The most common cognitive changes in aging are declines in memory, attention, and in information processing speed (Bashore et al., 1997). Slowing of processing speed as a significant contributor of age-induced changes in memory and attention may be documented with RT (Kail and Salthouse, 1994). Thus, the methodology of the study we did is relevant to the pathophysiologic changes typical of aging. On the plus side, we also have the fact that the tests are easy to administer.

### 5.1. Ideas Behind the Proposed Indices

#### 5.1.1. Index of Simple Reaction Time to Decision-Making Time (ISD)

Reaction time encompasses a series of subsequent processing transactions from encoding of a presented stimulus to a response execution. Unfortunately, RT does not reveal each of these transactions separately but gives a summary time length. Some researchers suggest using time latency of evoked potentials for measuring the transactions successively, one after another (Bashore et al., 1997). Our approach is to use a set of tasks with the following specific features. Instead of measuring RT in a single task or using disparate tasks, we constructed a battery of tests in such a way that the testing modalities (SVMR and CVMR) have the same perceptual and motor response components but differ in the central processing (DMT). This allows us to test the complexity hypothesis of James Birren et al., which says that neurocognitive slowing is restricted to the central nervous system processing and the amount of slowing increases when the level of the task complexity grows up (Birren et al., 1979, 1980). However, this is true for non-lexical tasks, whereas in the word processing tasks slowing does not correlate with the complexity (Bashore et al., 1997).

Our findings justify the strong version of the complexity hypothesis. According to the version, all the elements of information processing (e.g., perceiving, reasoning, and responding) slow to the same degree. The ISD index remains unchanged as the time spent on reasoning in CVMR retards across the years of life at the same pace as the summary length of receiving, encoding, and responding components. This makes the ratio between DMT and SVMR_mean stable throughout years on a population scale.

The strong version of the complexity hypothesis offers distinct advantages. It supports the idea that age-related retardation is associated with the general slowing in the processing speed rather than in components of information processing. It also vastly simplifies the tasks of neuroscientists searching for brain structure-functional associations while aging (Bashore et al., 1997). The justification of this point of view typically comes from the studies that, similar to our research, use RT as an aggregate measure of processing speed.

The weak version of the complexity hypothesis asserts that the level of a specific decline in perceptual, motor, decision, or attentional processes may be different. Some studies provide conflicting results, i.e., they show that “age-related slowing in simple repetitive tasks is mainly related to slowing at the stage of perceptuomotor processes, and after 60 years, to additional decline in attention” (Godefroy et al., 2010). Further research should utilize an event-related potentials technique to measure thoroughly the length of the transactions related to stimulus acquisition and response processing.

#### 5.1.2. Supplying ISD With Performance Accuracy (ISDA)

Decision-making time is the time of inhibition of an automatized action and task switching in CVMR test. The ratio of SVMR to DMT indicates the proportion between deciding and perceptive-motor components of the choice RT. The deciding and perceptive-motor components of CVMR have different cognitive loads. Comparing them, we see whether the age-related neuro cognitive retardation starts from the cognitively demanding acts (i.e., task switching) or involves both intellectual and non-intellectual functions (their generalized slowing).

**Figure 10:**
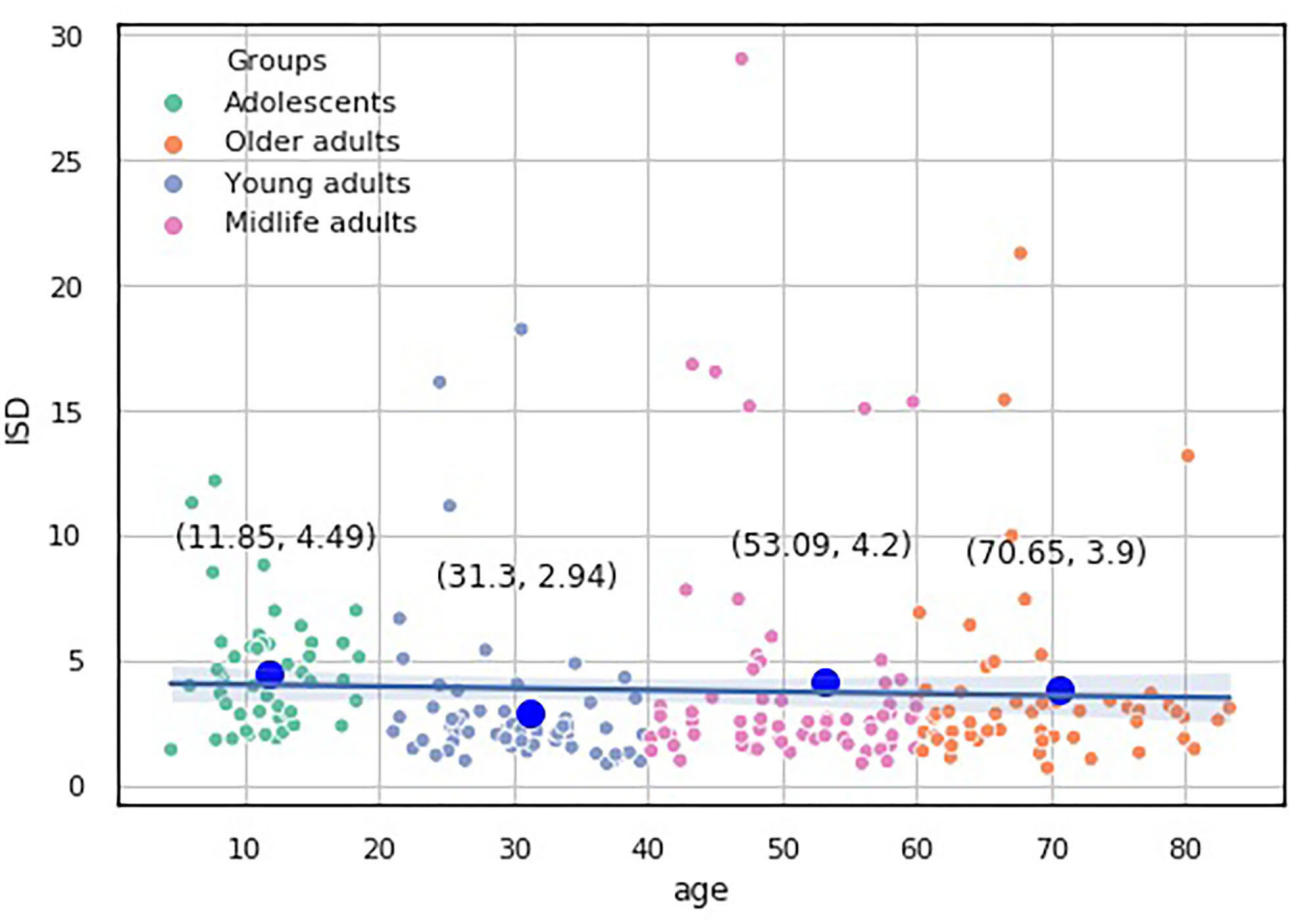
Over-the-age distribution of ISD values.

To get the overall efficiency of the examinee in the test, we supplied ISD index with the accuracy metric (see Figure 10). The idea behind the index is to inspect the proportion of the speed of processing of cognitively demanding to non-demanding tasks with the performance accuracy taken into consideration.

#### 5.1.3. Ratio of IES for Simple and Choice Reactions (ISCA)

The last index presents the IES ratio between simple and complex visual-motor reactions. IES score summarizes the overall efficiency of decision-making. IES accounts for different cognitive subdomains and may reflect their disproportional changes throughout the lifespan (Statsenko et al., 2020). We took into consideration that some studies documented differences between the lifelong changes of SVMR and CVMR (Der and Deary, 2006), but no study showed the same for IES scores. The possible explanation why this has not been done yet comes from the methodology of calculating IES score. To calculate the performance accuracy, neuroscientists mainly use the mistakes of choice made while performing “go/no-go” test (e.g., CVMR_false_reaction). The equipment that we used recorded two more types of mistakes: 1. missing the targeted events (e.g., SVMR_passes) and 2. preliminary responding (e.g., SVMR_falstart). This allowed us to calculate IES for the simple reaction and to compare it with the similar data for the choice reaction.

We consider that studying the association of RTs is less interesting than inspecting the relationship of IES for simple and choice reactions that are highly correlated. Simple RT accounts for 45% of variance of the choice RT (Der and Deary, 2006).

### 5.2. Age-Groups

To preselect and justify the age group ranges, we resorted to a heuristic approach and clustering. Then, we did a review to elucidate the biological changes that may underpin the choice of such subcohorts. The group boundaries correspond to the time points between the end of neurodevelopment, the appearance, and the acceleration of the cognitive decline. The following observations of other authors evidence this.

Healthy educated adults start developing age-related cognitive changes in their 20 and 30s (Salthouse, 2009). Reasonably, the age period before 20s is the time of massive neurodevelopment, acquiring skills, and possessing knowledge and intelligence.

In the next 20 years of life, cognitive functions undergo conflicting changes. Basic physiological cognitive functions decline early in life. Because of this, young adults may exhibit early signs of fluid intelligence, memory, and processing speed. At the same time, crystallized intelligence increases (Zimprich and Mascherek, 2010). In a study on simple RT, authors reported an increase in consistency of response with age from 8 to about 30, after which a decline starts. They observed the fastest response at the age of over 20 years, but the most consistent responding in terms of time variance comes at over 30 years (Pierson and Montoye, 1958). A roughly similar timeline of changes was received in another study: The shortest RT comes in the examinees' mid-20s (Rabbitt, 1993).

The summary volume of the brain white matter rises up until the early middle-age adulthood (aged over 35 year) (Ferreira et al., 2014; Nilsson et al., 2014). Then comes a period of stability in terms of WM volume and cognitive performance (Ferreira et al., 2014; Nilsson et al., 2014). At this period of life, neurocognitive slowing may impair cognitive abilities of midlife adults. Neuroplasticity stimulated by physical and mental exercising may attenuate the changes and ameliorate the cognitive status (Bauermeister and Bunce, 2016; Haynes et al., 2017). Nonetheless, cognitive decline is already evident in the middle-aged population (Singh-Manoux et al., 2012). But its more accurate onset remains an issue of debate (Singh-Manoux et al., 2012).

Accelerated cognitive decline starts only after the late middle age (55–60 years) (Ferreira et al., 2014; Nilsson et al., 2014). It is marked with a massive WM volume reduction, while gray matter volume follows a steady rate of reduction throughout life.

### 5.3. Estimates of the Dis/Proportional Changes in Cognitive Domains

Our findings suggest that there are proportional age-related changes in the time estimates of inhibitory control in task switching (e.g., DMT) and information processing speed (e.g., SVMR_mean). We claim that this is a feature of normal brain aging because we did a cross-sectional study of the healthy participants. In accelerated brain aging, the pattern of the changes may differ, in which case the proposed indices may serve as a screening tool for detecting such conditions. To verify this, a comparison study of the cohort of patients with dementia is required.

We do not see a significant accuracy decline in the relative CI of the performance of RMO technique across the lifespan. This is consistent with the origins of the Cognitive Aging Theory according to which intelligence does not decline with age. At that time, this pushed neuroscientists to correct the intelligence test data for the speed of processing (Anderson and Craik, 2017). Analyses revealed that most of the age differences in the cognitive performance were attributable to a slower speed of executing a relevant operation (Salthouse and Fristoe, 1995). Our recent studies conducted with the same battery of tests also showed that the age-related decline in reaction time in the attention study test is the most prominent feature of neurocognitive decline.

Many studies of the speed of processing have failed to develop unconditioned criteria that could help to distinguish normal aging from abnormal brain changes. Age-related declines in measures of cognitive functioning have been known as relatively large. They begin in early adulthood, manifesting themselves in several different types of cognitive abilities (Salthouse, 2004). To gain a better insight into the issue, scientists should investigate the interrelated changes of the cognitive abilities. A study of the proportionality of changes in the interdependent cognitive domains may supply the missing evidence to build up a reliable screening test for patients with dementia.

### 5.4. Informative Value of the Index of Performance in Simple and Complex Visual-Motor Reaction With Account for Accuracy

We hypothesized that a single index (e.g., ISCA) may substitute the dependent variables of the battery of PTs. In this way, it may serve as a marker of the psychophysiological status. If so, machine learning algorithms are capable to calculate its value out of other PT results. The potential of PTs to predict the values of ISCA is illustrated in Table 5. The performance metrics are good; the proportion of MAE to the range of values is low (3.49 ± 0.14%). All the created regression models are quite reliable. Random Forest regressor showed the best performance (3.36%). The accuracy of the models is higher than that of the models that we built recently for predicting IES score out of PT data (3.36 – 3.77% vs. 3.37 – 5.15%) (Statsenko et al., 2020).

The predictive accuracy is almost equal in all the age-groups studied. In contrast to this, the performance of predicting IES varies regarding the age: It is maximal for Adolescents and a bit lower for Older adults (Statsenko et al., 2020). This reduces the reliability of IES and makes ISCA the most suitable index for assessing psychophysiological performance and comparing the results irrespective of the age of examinees. As it is hard to segregate between the normal vs. accelerated aging, the index which is not vulnerable to aging may improve the currently existing strategies for the early detection of dementia.

## 6. Strengths and Limitations of the Study

The known limitation of the study—a restricted number of participants—is quite common for research on the current issue. Generally, there is a trade-off between the number of participants involved in a study on aging and the accuracy of selecting examinees. The stricter the inclusion criteria are, the smaller the study cohort is. Because of this, studies of normal aging are limited either in the cohort size or in evidence. In population-scale studies, no fund can cover expenses for MRI which is a golden standard of non-invasive screening for early stages of dementia. To reduce the cost of research, some neuroscientists resort to low-strength magnetic field MRI (Taki et al., 2008). In our study, we worked out a balanced solution based on the utilization of a high-field MRI and a thorough selection of the study participants (see exclusion criteria in subsection 3.2). The thorough selection of the participants who could fit the inclusion criteria limited the size of the study cohort. At the same time, its analysis provided a level of evidence that is impossible to reach in a population-scale survey with less tough inclusion criteria (e.g., Der and Deary, 2006). Also, there are known studies on the issue with the number of participants smaller (Jernigan et al., 1990; Gur et al., 1991; Malko et al., 1991; Foundas et al., 1998; Resnick et al., 2000; Scahill et al., 2003; Li et al., 2004; Edsbagge et al., 2011) or almost similar to our research (Pierson and Montoye, 1958; Grieve et al., 2005).

On the plus side, we have a big range of the age of the participants and their equal distribution over the time scale. This allows us to build the plots covering the entire population without the approximation for years. Unfortunately, some research concentrated on timing the onset of cognitive decline and many longitudinal studies miss the subjects younger than middle age (Singh-Manoux et al., 2012). Studies with a larger number of participants commonly fail to present people of all the age categories in equal proportion (Coffey et al., 1992; Good et al., 2001; Chen et al., 2007).

We did not take into consideration the education level of the participants unless they met the inclusion criteria (e.g., literate). There is no agreement on the issue in the literature as well. While some authors counted the number of years of full education, other researchers justified that education slows the decline of crystallized intelligence rather than other cognitive abilities. This is why a lower educational level is not predictive of a decline of the cognitive speed, memory, or reaction time in tests (Christensen et al., 1997). So, the absence of control of years of education cannot be considered a limitation of this study that deals with the reaction time and accuracy.

## 7. Conclusion

- Unsupervised ML clustering shows that the optimal number of homogeneous age-groups is four. We segregated the study sample into groups with the age range of 20 years starting from birth: Adolescents ∈ [0, 20), Young adults ∈ [20, 40), Midlife adults ∈ [40, 60), and Older adults ≥ 60 year of age.
- Our findings justify the strong version of the complexity hypothesis. The version assumes that all the elements of information processing (e.g., perceiving, reasoning, and responding) slow to the same degree. The ISD index remains unchanged as the time spent for reasoning in CVMR retards across the years of life with the same pace as the summary length of the receiving, encoding, and responding components. This makes the ratio between DMT and SVMR_mean stable throughout years on the population scale.
- To extend the utility of machine learning classification in cognitive studies, we proposed the estimates of the disproportional changes in cognitive functions (ISD, ISDA, and ISCA). The distribution of the indices and the values of the RMO test over the age provide the evidence that diverse cognitive functions decline equally throughout life.
- The ISD, ISDA, and ISCA values are more stable across the lifespan than the major dependent variable of the battery of the psychophysiological tests we used. After neurodevelopment and maturation, the indices preserve almost constant values with a slight trend toward functional decline. A new study is required to determine the utility of the ratio for segregating the normal brain aging from the accelerated one.
- The results of other psychophysiological tests (e.g., the accuracy, reaction time, and its variance in the simple visual-motor task, “go/no-go” test and “reaction to a moving object” test) did not demonstrate any general trend over age.
- The ISCA index for PT proved to be reliable: It reflects the overall psychophysiological status of an individual. We predicted ISCA values out of the results of other psychophysiological tests with high accuracy (mean absolute error/index range was 3.49 ± 0.14% vs. 7.62 ± 0.5% in ISD and 7.57 ± 0.55% in ISDA; *p* < 0.05). The accuracy of the models is higher than that of the models that we built recently for predicting IES score out of PT data (3.36−3.77% vs. 3.37−5.15%). In contrast to the models predicting IES score, the prediction performance on ISCA does not vary regarding the age. This reduces the reliability of IES and makes ISCA the most suitable index for assessing psychophysiological performance and comparing the results disregarding the age of examinees. As it is hard to segregate between the normal vs. accelerated aging, the index that is not sensitive to aging may improve the currently existing strategies for the early detection of dementia.
- In normal brain aging, there are proportional age-related changes in the time estimates of information processing speed and inhibitory control in task switching. Future research is supposed to collect the test data for the patients with dementia to determine whether the changes in the aforementioned indicators follow a different pattern.

## Data Availability Statement

The datasets presented in this study can be found in online repositories. The datasets generated for this study are available on request at the site of Big Data Analytics Center (BIDAC) at https://bi-dac.com.

## Ethics Statement

The study underwent ethical review by UAEU Human Research Ethics Committee (Notice Number: ERH_2019_4006 19_11) and got approval for the retrospective analysis of the data obtained as a standard of care. Written patient's consent or parental consent with assent from minors for being tested and scanned was obtained in each case.

## Author Contributions

All authors contributed to the creation of the article as follows. YS and TH contributed to the conceptual idea of the manuscript. YS formulated the objectives and wrote the manuscript. TH performed the statistical analysis and prepared the figures and tables for data presentation, and illustration. KN-VG with NZ, TA, FA, ML, and MB contributed to the literature review and data analysis.

## Funding

This work was supported by Aspire grant AARE19-060 (PI: ML) and UAEU StartUp grant 31M442 (PI: YS).

## Conflict of Interest

The authors declare that the research was conducted in the absence of any commercial or financial relationships that could be construed as a potential conflict of interest.

## Publisher's Note

All claims expressed in this article are solely those of the authors and do not necessarily represent those of their affiliated organizations, or those of the publisher, the editors and the reviewers. Any product that may be evaluated in this article, or claim that may be made by its manufacturer, is not guaranteed or endorsed by the publisher.

## Abbreviations

ACC: Accuracy
CVMR: complex visual-motor reaction
DMT: decision-making time
EF: executive functioning
EFT: executive functioning test
IES: inverse efficiency score
ISCA: index of performance in simple and complex visual-motor reaction with account for accuracy
ISD: index of simple reaction time to decision-making time
ISDA: index of simple reaction time to decision-making time with the accuracy performance
LR: Linear regression
ML: machine learning
PS: psychophysiological status
PT: psychophysiological test
RMO: reaction to a moving object
ROC AUC: Receiver Operating Characteristic Area Under the Curve
SVM: Support Vector Machine
SVMR: simple visual-motor reaction
Sens.: Sensitivity
Spec.: Specificity
TRVI: the time delay in responding to the targeted stimulus because of visual interfering objects
WM: white matter.
