# Supplementary material for: Proportional Changes in Cognitive Subdomains During Normal Brain Aging
Source: Front Aging Neurosci. 2021 Nov 15;13:673469. doi: 10.3389/fnagi.2021.673469 (PMC8634589; doi:10.3389/fnagi.2021.673469)
Supplement: Supplementary file 1 [file Data_Sheet_1.PDF]

SUPPLEMENTAL MATERIAL

Table 1. Characteristics of participants of the study

**Variable**	**Total** n_1=231	**Female** n_2=134 (58.01%)	**Male** n_3=97 (41.99%)	p_{2-3}
Age	40.96 [24.87-59.76]	47.3±20.08	33.73±21.98	**<0.0007**
Adolescents	48 (20.78%)	19 (14.18%)*	29 (29.9%)*	
Young adults	64 (27.71%)	36 (26.87%)	28 (28.87%)	**<0.0078**
Midlife adults	64 (27.71%)	39 (29.1%)	25 (25.77%)	
Older adults	55 (23.81%)	40 (29.85%)*	15 (15.46%)*	

* If the proportion of males and females in an age group is significantly different compared to other groups, such the group is marked with an asterisk.

Table 2. Predictors used to build machine learning models

**Predictor**	**Model outcome**		
	**Classification by age group**	**Regression predicting**	
		**ISD and ISDA indices**	**ISCA index**
Index	ISD / ISDA / ISCA		
Age		+	+
DMT	+		
TRVI	+	+	+
SVMR_mean	+		
SVMR_variance	+	+	+
SVMR_mistakes	+		
SVMR_IES	+	+	
CVMR_mean	+		
CVMR_variance	+	+	+
CVMR_mistakes	+		
CVMR_IES	+		
AST_mean	+	+	+
AST_variance	+	+	+
AST_errors	+	+	+
AST_IES	+	+	+
IRT_mean	+	+	+
IRT_variance	+	+	+
IRT_errors	+	+	+
IRT_IES	+	+	+
RMO_mean	+	+	+
RMO_variance	+	+	+
RMO_errors	+	+	+
WDL_MMS	+	+	+
WDL_MMS_inverse	+	+	+
WDR_MMS	+	+	+
WDR_MMS_inverse	+	+	+
AC	+	+	+
